# TRM4 is essential for cellulose deposition in Arabidopsis seed mucilage by maintaining cortical microtubule organization and interacting with CESA3

**DOI:** 10.1111/nph.15442

**Published:** 2018-09-13

**Authors:** Bo Yang, Cătălin Voiniciuc, Lanbao Fu, Sabine Dieluweit, Holger Klose, Björn Usadel

**Affiliations:** ^1^ Institute for Botany and Molecular Genetics (IBMG) BioEconomy Science Center RWTH Aachen University 52056 Aachen Germany; ^2^ Institute for Bio‐ and Geosciences (IBG‐2: Plant Sciences) Forschungszentrum Jülich 52425 Jülich Germany; ^3^ Institute of Complex Systems (ICS‐7) Forschungszentrum Jülich 52425 Jülich Germany

**Keywords:** *Arabidopsis*, cellulose deposition, cellulose synthase (CESA), microtubule organization, mucilage, TONNEAU1 recruiting motif (TRM)

## Abstract

- The differentiation of the seed coat epidermal (SCE) cells in *Arabidopsis thaliana* leads to the production of a large amount of pectin‐rich mucilage and a thick cellulosic secondary cell wall. The mechanisms by which cortical microtubules are involved in the formation of these pectinaceous and cellulosic cell walls are still largely unknown.
- Using a reverse genetic approach, we found that TONNEAU1 (TON1) recruiting motif 4 (*TRM4*) is implicated in cortical microtubule organization in SCE cells, and functions as a novel player in the establishment of mucilage structure.
- TRM4 is preferentially accumulated in the SCE cells at the stage of mucilage biosynthesis. The loss of *TRM4* results in compact seed mucilage capsules, aberrant mucilage cellulosic structure, short cellulosic rays and disorganized cellulose microfibrils in mucilage. The defects could be rescued by transgene complementation of *trm4* alleles. Probably, this is a consequence of a disrupted organization of cortical microtubules, observed using fluorescently tagged tubulin proteins in *trm4* SCE cells. Furthermore, TRM4 proteins co‐aligned with microtubules and interacted directly with CELLULOSE SYNTHASE 3 in two independent assays.
- Together, the results indicate that TRM4 is essential for microtubule array organization and therefore correct cellulose orientation in the SCE cells, as well as the establishment of the subsequent mucilage architecture.

## Introduction

The seed coat is essential for the protection of the embryo during embryogenesis and the facilitation of seed dispersal, dormancy and germination (Windsor *et al*., 2000). In *Arabidopsis thaliana*, fertilization initiates the differentiation of the seed coat epidermal (SCE) cells, which are derived from the outermost layer of the outer ovule integuments (Haughn & Chaudhury, 2005). The SCE cells undergo dramatic changes during development, including the sequential deposition of two types of specialized cell wall (Voiniciuc *et al*., 2015c). The synthesis and secretion of plentiful pectinaceous mucilage is followed by the production of a thick cellulose‐rich wall, known as the columella (Western, 2006). On exposure to water, the hydrophilic mucilage ruptures the primary wall and envelops the seed, forming a sticky two‐layered mucilage capsule that contains cellulosic ray‐like structures. The non‐adherent layer of mucilage is diffuse and can easily be detached from the seed by gently shaking in water. The adherent layer of mucilage is strongly associated with the seed and can be removed by vigorously shaking in water, strong acid, strong base or cell wall hydrolytic enzyme treatments (Haughn & Western, 2012; Voiniciuc *et al*., 2015c). The major component of both pectinaceous layers is rhamnogalacturonan I (RG I), but small amounts of other polymers have also been detected in adherent mucilage: homogalacturonan (HG; Macquet *et al*., 2007), cellulose (Harpaz‐Saad *et al*., 2011; Mendu *et al*., 2011; Sullivan *et al*., 2011), galactoglucomannan (Yu *et al*., 2014; Voiniciuc *et al*., 2015b) and xylan (Voiniciuc *et al*., 2015a; Hu *et al*., 2016a,b; Ralet *et al*., 2016).

Despite being a minor component in mucilage, cellulose plays an essential role in reinforcing the columella of SCE cells (Stork *et al*., 2010) and anchoring the mucilage pectic components to the seed (Harpaz‐Saad *et al*., 2011; Mendu *et al*., 2011; Sullivan *et al*., 2011). The cellulose microfibril consists of hydrogen‐bonded linear β‐1,4‐glucan chains (Kimura *et al*., 1999) which are synthesized by rosette‐shaped CELLULOSE SYNTHASE (CESA) complexes (CSCs) formed by three different catalytic subunits (Somerville *et al*., 2004). In *A. thaliana*, CESA1 and CESA3 are key subunits of the CSCs involved in primary wall cellulose synthesis, whereas CESA6‐like (CESA6, CESA2, CESA5 and CESA9) proteins are partially redundant in various developmental contexts (Desprez *et al*., 2007; Persson *et al*., 2007). Previous studies have shown that CESA3 and CESA5 are critical for cellulose biosynthesis in SCE cells and influence mucilage extrusion and adherence (Mendu *et al*., 2011; Sullivan *et al*., 2011; Griffiths *et al*., 2015; Griffiths & North, 2017). In addition, proteins that may be associated with the CSCs, such as COBRA‐LIKE 2 (COBL2), influence cellulose synthesis in the seed coat and mucilage adherence (Ben‐Tov *et al*., 2015, 2018). Although several genes involved in mucilage cellulose synthesis have been identified, the detailed mechanisms of microfibril synthesis, alignment and the additional elements associated with cellulose synthesis and deposition in SCE cells remain rather unclear.

Live‐cell imaging of fluorescently tagged CESAs and microtubule proteins indicates that cortical microtubules act as molecular rails along which the CSCs track during cellulose synthesis (Paredez *et al*., 2006). CESA3, CESA5 and CESA10 are highly expressed in SCE cells during the period of mucilage production and move in a linear and unidirectional manner around the cytoplasmic column of the SCE cells guided by microtubules (Griffiths *et al*., 2015). To date, only one microtubule‐associated protein, MICROTUBULE ORGANIZATION 1 (MOR1), has been implicated to play a role in mucilage extrusion (McFarlane *et al*., 2008). The data from previous studies support the hypothesis that microtubules guide the CSCs to deposit cellulose in the mucilage pockets.

Microtubules typically interact with many different proteins to fulfill their roles in the physiology of cells. For instance, *TONNEAU1* (*TON1*) encoding proteins, similar to human FOP centrosomal proteins, and *FASS*/*TON2* encoding phosphatase 2A (PP2A) regulatory B subunit proteins are essential for the organization of microtubule arrays (Camilleri *et al*., 2002; Azimzadeh *et al*., 2008). TON1 recruiting motif (TRM) proteins can target TON1 and FASS/PP2AB proteins to the microtubules and form a TON1/TRM/PP2A regulatory complex called TTP (Drevensek *et al*., 2012; Spinner *et al*., 2013). The TTP complex is implicated in the formation of the preprophase band (PPB) and the organization of interphase cortical microtubules (Spinner *et al*., 2013). Thus, TRM proteins are required for the recruitment of the TTP complex to the cortical microtubules and play important roles in the maintenance of microtubule organization (Spinner *et al*., 2013). A recent study has also revealed that TRM6, TRM7 and TRM8 participate in PPB formation required for the orientation of plant cell division (Schaefer *et al*., 2017).

In this study, we identified TONNEAU1 (TON1) recruiting motif 4 (*TRM4*) as a player in mucilage synthesis through coexpression studies. *TRM4* loss of function leads to aberrant orientation of cellulose microfibrils and restrained cellulosic ray length in mucilage, which results in a mucilage extrusion defect. TRM4 proteins co‐localize with microtubules, interact with CESA3 proteins and are crucial for cortical microtubule organization in SCE cells. Therefore, we propose that TRM4 plays important roles in mucilage cellulose deposition, cellulosic ray length and the establishment of mucilage architecture.

## Materials and Methods

### Plant materials

The *Arabidopsis thaliana* Col‐0 wild‐type (WT) plant and mutant lines (*trm4‐1*, SALK_022851; *trm4‐2*, SALK_068678; *trm4‐3*, GK‐821C08; *trm3‐1*, GK‐316D12; *trm3‐2*, SALK_127245; *trm4‐3 trm3‐1*, GABI DUPLO Double Mutant 195; *muci10‐1*, SALK_061576; *csla2‐3*, SALK_149092; *ixr1‐2*, ethylmethane sulfonate line, NASC number: N6202) were obtained from the Nottingham Arabidopsis Stock Centre. *cesa5‐*1 (Mendu *et al*., 2011), *muci10‐1* (Voiniciuc *et al*., 2015b), *csla2‐3* (Yu *et al*., 2014) and *ixr1‐2* (Griffiths *et al*., 2015) have been described previously. The *pUBQ:RFP‐TUB6* plant seeds were obtained from Dr Geoffrey Wasteneys (Ambrose *et al*., 2011). The plants were grown under constant light (120 μE m^−2^ s^−1^), 20°C and 60% humidity.

### Phylogenetic analysis

The 34 TRM protein sequences in *A. thaliana* and 26 putative TRM proteins in *Solanum lycopersicum* were retrieved as described previously (Wu, 2015). The six putative TRM protein sequences in *Oryza sativa* were obtained using the AtTRM4 sequence for blastp search in The Rice Annotation Project Database. The data were used for phylogenetic analysis in mega6.0 software (Tamura *et al*., 2013), as described previously (Voiniciuc *et al*., 2015a).

### RNA isolation and quantitative reverse transcription–polymerase chain reaction

RNA was isolated using a MasterPure Plant RNA Purification Kit (Epicentre, Madison, WI, USA). The RNA was used for first‐strand complementary DNA (cDNA) synthesis by M‐MLV Reverse Transcriptase (Promega). The cDNA was used as template for quantitative reverse transcription–polymerase chain reaction (qRT‐PCR) analysis with primers (Supporting Information Table S1). qRT‐PCR was carried out using a Platinum SYBR Green qPCR SuperMix‐UDG Kit (Invitrogen) in a Step One Plus Real‐Time PCR System (Applied Biosystems, Life Technologies, Marsiling Industrial Estate, Singapore).

### Construction of vectors

The *TRM4* promoter fragment (*pTRM4*), 2274‐bp upstream sequence from the *TRM4* coding sequence, was amplified using Phusion High‐Fidelity DNA Polymerase and introduced into the pPLV13:GUS vector by the ligation‐independent cloning (LIC) technique (De Rybel *et al*., 2011). The pTRM4:TRM4‐YFP construct was created based on the pCV01 vector 35S:LIC‐YFP (Voiniciuc *et al*., 2015b). The TRM4 cDNA fragment was amplified and introduced into pCV01. *pTRM4* was introduced into pCV01 using *Kpn*I and *Apa*I. The p35SS:YFP‐TRM4, pUBQ10::GFP‐TRM4 and pUBQ10::RFP‐TUB6 constructs were generated using Gateway technology. The TRM4F1/2/3 fragments were introduced into the pTRAkt vector p35SS:GFP by *Eco*RI and *Pci*I. The full‐length cDNA of the gene of interest (CESA3, TRM4, TRM26 and CC1) was introduced into the yeast two‐hybrid (Y2H) vectors (pMet‐Cub‐R‐Ura3 for use as bait vector and pNX32_GW for use as prey vector) and the bimolecular fluorescence complementation (BiFC) vectors (pUBN‐cYFP‐Dest and pUBN‐nYFP‐Dest) by Gateway technology. The cloning and sequencing primers are listed in Table S1.

### β‐glucuronidase staining of plant tissues

5‐Bromo‐4‐chloro‐3‐indolyl glucuronide (X‐Gluc) was used as substrate for β‐glucuronidase (GUS) staining. Plant tissues were applied in a vacuum in X‐Gluc solution containing 39 mM NaH_2_PO_4_, 61 mM Na_2_HPO_4_, 2 mM K_3_Fe(CN)_6_, 0.5 mg ml^−1^ X‐Gluc and 0.1% Triton‐X100. The samples were incubated for 1–3 d at 37°C. Then, the samples were incubated in 70% ethanol for de‐staining for 3 d. The de‐stained plant tissues were incubated in 50% glycerol for 2 d and observed using a Leica MZ12 stereomicroscope (Wetzlar, Germany).

### Ruthenium red staining and quantification of mucilage area

Seeds were hydrated with water, 50 mM CaCl_2_ or 50 mM ethylenediaminetetraacetic acid (EDTA), pH 9.5, for 60 min at 125 rpm on an orbital shaker. The seeds were stained with 0.01% (w/v) Ruthenium red (RR) for 20 min and imaged using a stereomicroscope. The quantification of mucilage and seed areas was analyzed using ImageJ as described previously (Voiniciuc *et al*., 2015b).

### Other histological techniques

For Pontamine fast scarlet 4B (S4B) cellulose staining, hydrated seeds were stained with 0.01% (w/v) S4B (Sigma‐Aldrich) in 50 mM NaCl for 60 min (Mendu *et al*., 2011). The lengths of cellulosic rays were measured by ImageJ. Calcofluor staining was performed as described by Willats *et al*. (2001). Fluorescein isothiocyanate (FITC)‐dextran staining was performed as described previously (Voiniciuc *et al*., 2015b).

### Immunolabeling assays

CBM3a immunolabeling was performed as described previously (Voiniciuc *et al*., 2015b). The mucilage was immunolabeled with CCRC‐M30 as described previously (Voiniciuc, 2017; Voiniciuc *et al*., 2015a). Images were acquired with a Leica SP8 confocal microscope: calcofluor signal (405 nm excitation and 410–452 nm emission); S4B signal (561 nm excitation and 600–650 nm emission); Alexa Fluor signal (488 nm excitation and 500–550 nm emission).

### Enzyme‐linked immunosorbent assay (ELISA) of CBM3a

Seeds (4 mg) were precisely weighed and the total mucilage was extracted by shaking in 1 ml of water for 30 min at 30 Hz using a Retsch mill; 200 μl of mucilage extract were transferred to four wells on a 96‐well ELISA plate (3598; Corning, Wiesbaden, Germany); 200 μl of water served as a negative control. The wells were dried and blocked with 200 μl of blocking solution (1% bovine serum albumin (BSA) in phosphate‐buffered saline (PBS), pH 7.0) at 125 rpm for 30 min. Then, 5 μl of histidine (His)‐tagged CBM3a and 45 μl of 0.1% w/v BSA in PBS were added to each well and incubated at 125 rpm for 60 min. The wells were sequentially incubated with 50 μl of the primary monoclonal antibody (mAb) anti‐His mouse antibody (Sigma‐Aldrich), diluted 1 : 1000 in antibody solution, and 50 μl of the secondary mAb peroxidase‐conjugated goat anti‐mouse IgG antibody (Sigma‐Aldrich), diluted 1 : 1000 in 0.1% w/v BSA in PBS, at 125 rpm for 60 min. Then, the wells were washed five times with PBS. 3,3′,5,5′‐Tetramethylbenzidine substrate solution (50 μl) (Sigma‐Aldrich) was added and incubated at 125 rpm for 20 min. The reaction was stopped by the addition of 50 μl of 0.5 M sulfuric acid. The optical density (OD) value was read as the difference between the absorption value at 450 nm and 655 nm using a Synergy H1 microplate reader (BioTek, Bad Friedrichshall, Germany). The reading from each test well was subtracted from that of the negative control well.

### Crystalline cellulose observation and content determination

Birefringence of crystalline cellulose and crystalline cellulose content quantification in mucilage were examined as described previously (Voiniciuc *et al*., 2015b). The crystalline cellulose content was determined by the Updegraff assay (Updegraff, 1969).

### Mucilage extraction and monosaccharide composition analysis

The non‐adherent mucilage was extracted by shaking horizontally in an orbital shaker at 200 rpm for 90 min. The total mucilage was extracted by vigorous shaking in a Retsch mill at 30 Hz for 30 min, and the monosaccharide composition was analyzed as described previously (Voiniciuc *et al*., 2015b; Voiniciuc & Günl, 2016).

### Glycosyl hydrolase treatments

Seeds were imbibed in 500 μl of 0.1 M sodium acetate buffer, pH 4.5, with or without 10 units of endo‐1,4‐β‐d‐glucanase (*Trichoderma longibrachiatum*, E‐CELTR; Megazyme, Wicklow, Ireland). For high‐purity recombinant cellulase (*Bacillus amyloliquefaciens*, E‐CELBA; Megazyme) treatment, seeds were hydrated with 500 μl of 0.1 M phosphate buffer, pH 6.0, consisting of 1 mg ml^−1^ BSA, with or without 10 units of the enzyme. Plates were incubated at 125 rpm for 90 min at 37°C. Finally, the buffers and cellulases were removed and rinsed once with water before RR or S4B staining.

### Scanning electron microscopy

The samples were sputter coated with a gold layer (*c*. 5 nm thickness, 60 mA current) using a Cressington Sputter Coater 208 HR integrated with a thickness controller MTM‐20 (Cressington Scientific Instruments Ltd, Watford, UK). Several seeds of each sample were mounted on a typical electron microscopy stub using a carbon adhesive tape. The scanning electron microscopy (SEM) images were acquired using a LEO 1550 field emission SEM (Zeiss, Oberkochen, Germany) with an in‐lens or secondary electron detector at 5–15 kV acceleration voltage in the cleanroom of the Helmholtz Nano Facility (Albrecht *et al*., 2017).

### Yeast split‐ubiquitin two‐hybrid analysis

The split‐ubiquitin assay was performed in *Saccharomyces cerevisiae* strain JD53. Briefly, bait and prey vectors were transformed into yeast cells as described previously (Gietz & Schiestl, 2007). Transformants were selected on synthetic complete medium minus histidine and tryptophan (SC‐HT) with and without 1 mg ml^−1^ 5‐fluoroorotic acid (5‐FOA) for 5 d. To quantify the interactions, 80 colonies of each combination were spotted onto SC‐HT medium with 5‐FOA and grown at 30°C for 3 d. The number of spots grown was then counted (Vain *et al*., 2014).

### Plant transformation and analysis of fluorescent protein localization

*Arabidopsis thaliana* was transformed using the floral dip method (Clough & Bent, 1998). BiFC and transient expression were performed in *Nicotiana benthamiana* leaves as described previously (Grefen *et al*., 2010). The quantification of the BiFC experiment was performed as described previously (Kudla & Bock, 2016). For oryzalin treatment, small pieces of transfected leaf were cut and treated with 1% (v/v) dimethylsulfoxide (DMSO) as mock treatment or 1% (v/v) DMSO containing 0.1 mM oryzalin (Sigma‐Aldrich) for 12 h. For the observation of hypocotyl epidermal cells, the epidermal cells in zone 1 of 4‐d‐old dark‐grown seedlings were examined as described previously (Crowell *et al*., 2011). Microtubule angles were measured against the growth axis and in a clockwise direction by the angle tool in ImageJ (Bringmann *et al*., 2012b). For the observation of SCE cells, fully opened flowers were marked and the seeds were dissected from developing siliques at 7 d post‐anthesis (DPA). All the samples were mounted on microscope glass slides with spacers. Fluorescence was examined using a Leica SP8: green fluorescent protein (GFP) signal (488 nm excitation and 505–530 nm emission), yellow fluorescent protein (YFP) signal (514 nm excitation and 520–550 nm emission) and red fluorescent protein (RFP) signal (561 nm excitation and 575–635 nm emission). Intensity plot analysis was performed using Leica Suite X with intensity tool. The maximum projection of the *Z*‐stack view was generated by five frames using the Z Project tool with the maximum intensity projection type in ImageJ.

## Results

### Expression and phylogenetic analysis of *TRM4*

**Figure 1:**
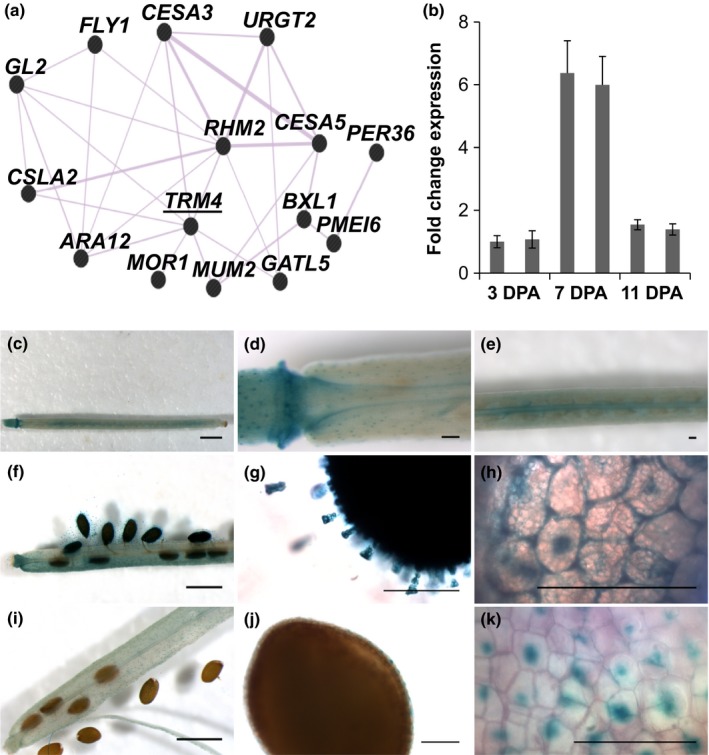
Expression analysis of TONNEAU1 (TON1) recruiting motif 4 (*TRM4*). (a) Co‐expression network of *TRM4* with 14 known mucilage genes based on Genemania (Warde‐Farley *et al*., 2010). GL2, Glabra2, a representative HD‐Zip transcription factor; FLY1, Flying saucer 1; ARA12, Subtilisin‐like protease; MUM2, Mucilage‐modified 2; PMEI6, Pectin methylesterase inhibitor 6; BXL1, Beta‐xylosidase 1; PER36, Peroxidase 36; CSLA2, Cellulose synthase‐like A2; URGT2, UDP‐l‐Rha/UDP‐d‐Gal transporter 2; CESA3/CESA5, Cellulose synthase 3/5; RHM2, Rhamnose biosynthase 2; GATL5, Galacturonosyl transferase‐like 5; MOR1, Microtubule organization 1. (b) Quantitative reverse transcription‐polymerase chain reaction (qRT‐PCR) analyses of *TRM4* expression in developing *Arabidopsis thaliana* siliques in the wild‐type (WT). Two WT biological replicates were examined at three stages of seed development (3, 7 and 11 d post‐anthesis (DPA), which are equivalent to pre‐globular, linear cotyledon and mature green stages, respectively) (Francoz *et al*., 2015). *TRM4* expression (normalized to UBIQUITIN10) relative to the first wild‐type in each set. Data show means ± SD of three technical replicates. (c–k) β‐Glucuronidase (GUS) activity in developing siliques and seed coat under the control of the *TRM4* promoter. (c–e) Silique at 3 DPA. (f–h) Silique and seed coat epidermal cells at 7 DPA. (i–k) Silique and seed coat epidermal cells at 11 DPA. Bars: (c, f, i) 1000 μm; (d, e, g, h, j, k) 100 μm.

Based on elastic net coexpression analysis with eight known mucilage genes (*RHM2*,* MUM2*,* ARA12*,* PMEI6*,* FLY1*,* BXL1*,* GL2* and *GATL5*) as bait, using an Affymetrix‐based microarray dataset comprising a seed and silique developmental series compiled by AtGenexpress (Schmid *et al*., 2005; Zou & Hastie, 2005), we found *TRM4* (AT1G74160) to be coexpressed with *RHM2* and *ARA12/SBT1.7* (Usadel *et al*., 2004; Western *et al*., 2004; Rautengarten *et al*., 2008). Using the Genemania tool (Warde‐Farley *et al*., 2010), *TRM4* was also predicted to be coexpressed with multiple mucilage genes (Fig. 1a). Seed microarray data visualized in the eFP browser (Winter *et al*., 2007; Le *et al*., 2010) revealed that the expression of *TRM4* peaks in the general seed coat of linear cotyledon and maturation green stages (Fig. S1a). This coincides with the synthesis of mucilage polysaccharides at the linear cotyledon stage, *c*. 7–10 DPA during seed development (Francoz *et al*., 2015; Voiniciuc *et al*., 2015c). Consistent with the microarray data, qRT‐PCR analyses in developing siliques showed that *TRM4* transcript peaks in WT siliques at 7 DPA, which is equivalent to the linear cotyledon stage (Fig. 1b).

To further characterize the transcriptional profile of *TRM4*, a *pTRM4:GUS‐fusion* construct was generated and stably transformed in WT plants. The expression patterns of GUS activity were visualized histochemically. Notably, in the developing siliques, the GUS signal was detected in epidermal cells of the seed coat (Fig. 1c–k). The highest GUS activity was recorded in seeds at the 7‐DPA stage (Fig. 1f–h). In seeds at the 11‐DPA stage, GUS staining was still detected in the columella (Fig. 1i–k). The GUS activity analyses confirmed the *TRM4* expression pattern in SCE cells demonstrated by the eFP microarray data and qRT‐PCR analyses. GUS activity was also detected in leaves and roots of 5‐d‐old seedlings, hydathodes and guard cells of leaves, and receptacles, stigma, petals and filaments of flowers (Fig. S1b–g), but not in the tissues of negative controls (Fig. S1h–j). The expression of *TRM4* in guard cells was consistent with eFP browser data (Fig. S1k).

Within the *A. thaliana* genome, *AtTRM3* (AT1G18620) is the closest paralog of *AtTRM4*, sharing 62% amino acid identity (Fig. S2). Phylogenetic analysis of the whole *TRM* gene family revealed that *AtTRM4* and *AtTRM3* cluster together with *AtTRM1* and *AtTRM2* (Fig. S2). *SlTRM3/4* in *S. lycopersicum* and *OsGL7/GW7* in *O. sativa* belong to this group (Fig. S2). Four members of this group (*AtTRM1*,* AtTRM2*,* SlTRM3/4* and *OsGL7/GW7*) have already been functionally characterized as microtubule‐associated proteins involved in the regulation of cell elongation (Drevensek *et al*., 2012; S. Wang *et al*., 2015, 2015; Y. Wang *et al*., 2015, 2015; Wu, 2015).

### The *trm4* mutant seeds have smaller mucilage capsules

**Figure 2:**
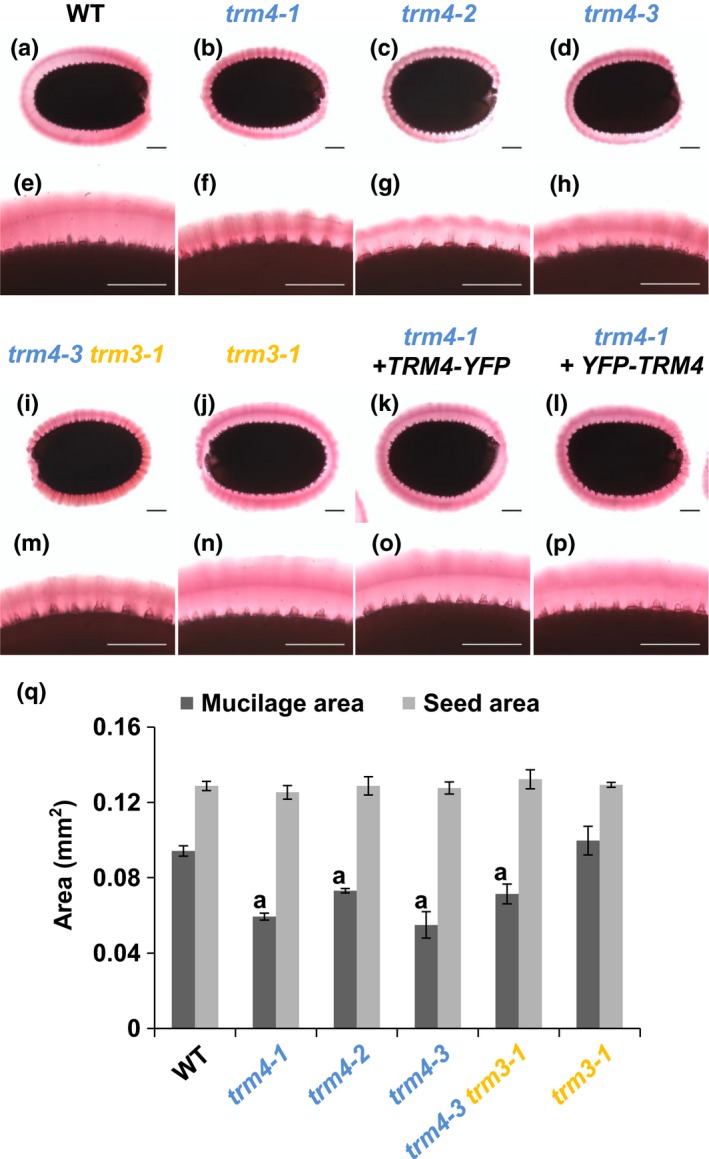
TONNEAU1 (TON1) recruiting motif 4 (*TRM4*) mutation leads to a compact mucilage capsule. (a–p) Ruthenium red (RR)‐stained seeds after hydration of wild‐type (WT) *Arabidopsis thaliana*, mutants and complementation lines driven by the *TRM4* and *P35SS* promoters. Bars, 100 μm. (q) Areas of seeds and RR‐stained mucilage capsules. Values represent means ± SD of five biological replicates (> 20 seeds each). The ‘a’ marks a significant decrease compared with WT (Student's *t*‐test, *P *<* *0.05).

In order to check whether TRM4 and TRM3 are implicated in mucilage establishment, we isolated three independent *trm4* single mutants, two *trm3* single mutants and a *trm4‐3 trm3‐1* double mutant (Fig. S3a). Based on qRT‐PCR analyses, all mutants showed severely reduced transcription of the affected genes (Fig. S3b). Relative to WT, RR staining revealed that the three *trm4* alleles and the *trm4‐3 trm3‐1* double mutant had a smaller mucilage capsule, whereas *trm3‐1* showed no obvious defects (Fig. 2). Quantification of mucilage areas revealed that *trm4* single mutants had *c*. 30% smaller capsules relative to WT (Fig. 2q), and that the *trm4‐3 trm3‐1* double mutant phenocopied the mucilage structure of the *trm4‐3* single mutant. Unlike the *trm4* mutants, the *trm3‐1* mucilage area resembled that of WT (Fig. 2q). An independent line, *trm3‐2*, showed similar results (data not shown). Microarray data revealed that *TRM3* is more predominantly expressed in leaves (Schmid *et al*., 2005). Hence, *TRM4* and *TRM3* do not play functionally redundant roles in mucilage establishment. In contrast with the mucilage dimensions, all mutants had seeds with a similar area to those of WT (Fig. 2q).

Transgene complementation was used to confirm that the *trm4‐1* mutation results in compact mucilage. In all complemented lines, qRT‐PCR analyses validated that *TRM4* expression was elevated to at least WT levels (Fig. S3b). Moreover, RR staining of seeds after hydration revealed that all complemented lines resembled WT mucilage capsules, indicating that the expression of *TRM4*, but not *TRM3*, was required for normal mucilage area (Figs 2, S4).

### TRM4 influences cellulosic ray length and cellulose deposition in seed mucilage

**Figure 3:**
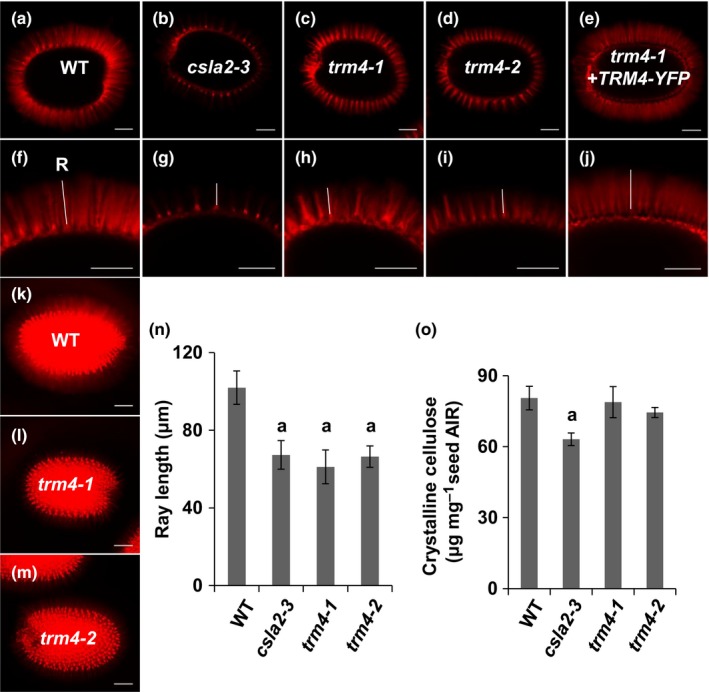
Cellulose deposition is altered in TONNEAU1 (TON1) recruiting motif 4 (*trm4*) mutants. (a–m) Pontamine fast scarlet 4B (S4B) cellulose staining. (k–m) Maximum projection of a *Z*‐stack of seeds with S4B cellulose staining. Bars, 100 μm. The white lines indicate the cellulosic rays (R) originating from the top of the columella. (n) Ray lengths measured by Image J from S4B staining images. (o) *Arabidopsis thaliana* seed crystalline cellulose quantified using the Updegraff assay AIR, alcohol insoluble residue. Values represent the means ± SD of (n) three biological replicates or (o) four biological replicates. The ‘a’ marks significant decreases compared with the wild‐type (WT) (Student's *t‐*test, *P *<* *0.05).

The compact mucilage phenotype was not associated with any significant changes in the monosaccharide composition of non‐adherent mucilage or total mucilage extracts from *trm4* alleles compared with WT (Table S2). Unlike previously identified mutants with compact mucilage, such as *csla2‐3* (Yu *et al*., 2014; Voiniciuc *et al*., 2015b), *trm4* seeds did not show any significant changes in crystalline cellulose content relative to WT (Fig. 3o). We then examined the precise distribution of cellulose in the mucilage capsule using S4B dye, which specifically binds cellulose (Anderson *et al*., 2010). WT mucilage capsules displayed ordered and intense S4B‐labeled cellulosic rays that projected outwards from the top of the columellae, as well as diffuse S4B signals between rays (Fig. 3a). By contrast, *trm4* seeds were surrounded by shorter cellulosic rays that looked like tufts, and lacked the diffuse S4B staining between rays (Fig. 3c,d). The maximum intensity z‐projection of the S4B signals across the seed surface revealed reduced cellulose distribution in *trm4* mucilage relative to WT (Fig. 3). The lengths of the S4B‐labeled rays in the mucilage capsules of the *trm4* mutants were *c*. 30% shorter than those of WT (Fig. 3n). These defects could be rescued by the expression of the *TRM4‐YFP* transgene in the *trm4‐1* mutant (Fig. 3e). In contrast with *trm4*, the previously described *csla2‐3* mutant seeds showed significant reductions in both crystalline cellulose content and S4B staining (Yu *et al*., 2014; Voiniciuc *et al*., 2015b). Although the *trm4* mutants resembled the shorter cellulosic ray length of *csla2‐3* seeds (Fig. 3n), they did not show lower S4B intensity or reduced seed crystalline cellulose content (Fig. 3o).

**Figure 4:**
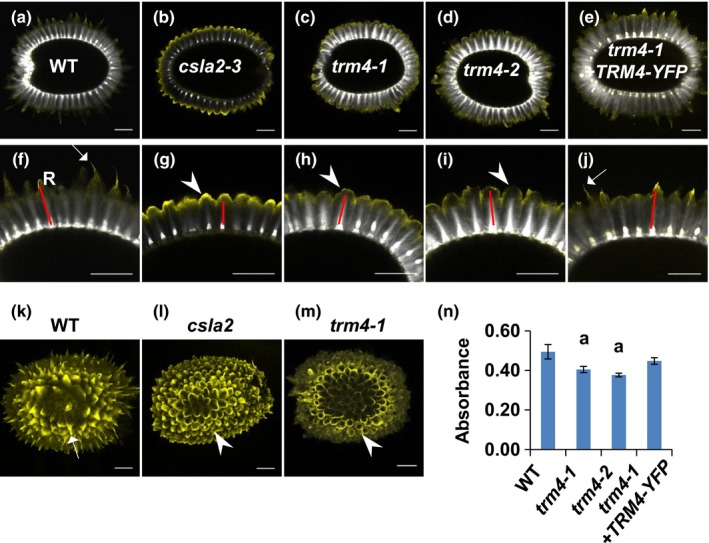
Immunolabeling of CBM3a (yellow) with high affinity to crystalline cellulose in adherent mucilage. (a–j) CBM3a immunolabeling with calcofluor (white) as counterstain. (k–m) Maximum projection of a *Z*‐stack of *Arabidopsis thaliana* seeds with CBM3a immunolabeling: (k) wild‐type (WT) seed; (l) *csla2* seed; (m) *trm4‐1* seed. (n) CBM3a enzyme‐linked immunosorbent assay (ELISA) showed decreased CBM3a epitopes in TONNEAU1 (TON1) recruiting motif 4 (*trm4*) mucilage cellulose. The *y*‐axis denotes the CBM3a ELISA‐corrected absorbance. The red lines indicate the cellulosic rays (R). Arrows highlight the mustache tip‐like structures. Arrowheads mark the mushroom cap‐like structures. Values represent means ± SD of four biological replicates. Bars, 100 μm. The ‘a’ marks significant decreases compared with WT (Student's *t‐*test, *P *<* *0.05).

To further characterize the fine structure of cellulose in seed mucilage, we used the CBM3a carbohydrate‐binding module, which detects crystalline cellulose in plant material (Blake *et al*., 2006), and calcofluor dye, which binds β‐glucans (Willats *et al*., 2001). As CBM3a immunolabeling and calcofluor staining of mucilage do not completely overlap, they can both be used to further delineate mucilage cellulosic structure. Similar to *csla2,* the CBM3a immunolabeling of *trm4* seeds displayed mushroom cap‐like structures on top of the mucilage rays (Fig. 4b–d). By contrast, WT and the *trm4‐1* complemented line showed mustache tip‐like structures on top of the mucilage rays (Fig. 4a,e). The maximum projection of the *Z*‐stack view of CBM3a‐labeled seeds clearly exhibited the mustache tip‐like structures on the top of WT mucilage rays, and the mushroom cap‐like structures on the top of *csla2* and *trm4* mucilage rays (Fig. 4k–m). ELISA of mucilage extracts demonstrated that the number of CBM3a epitopes was decreased by *c*. 18% in both *trm4* mutants relative to WT and the *trm4‐1* complemented line (Fig. 4n). CBM3a immunolabeling and ELISA indicate that *trm4* mutations decrease the crystallinity of mucilage cellulose and reduce its distribution.

### The alignment of mucilage cellulose microfibrils is disordered in *trm4* mutants

**Figure 5:**
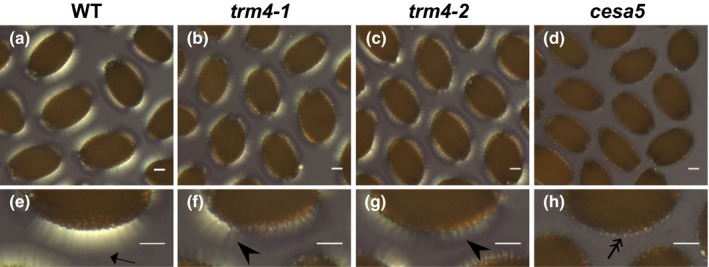
Disruption of TONNEAU1 (TON1) recruiting motif 4 (*TRM4*) leads to disordered alignment of mucilage cellulose microfibrils in *Arabidopsis thaliana*. (a–h) Birefringence of crystalline cellulose in mucilage. Arrow marks bright region; arrowheads mark region of impaired and disordered cellulosic rays; double arrow marks bright spot region. Bars, 100 μm. WT, wild‐type.

The highly ordered microfibrils in crystalline cellulose can produce birefringent mucilage capsules under polarized light (Sullivan *et al*., 2011). As CBM3a immunolabeling revealed altered cellulose spatial organization in *trm4* mucilage, we also examined the birefringence of seed mucilage cellulose. WT seeds were surrounded by large crystalline regions with ray‐like structures (Fig. 5), whereas *trm4* seeds showed impaired and patchy birefringent regions containing short and disorganized rays (Fig. 5). The cellulose‐deficient *cesa5* seeds lacked the crystalline rays and displayed some residual birefringence on the seed surface (Fig. 5). This implies that the alignment of crystalline cellulose microfibrils is disordered in *trm4* adherent mucilage.

### Cellulase treatments confirm the altered cellulose properties in *trm4*

The aberrant architecture of *trm4* mucilage was further examined by enzymatic digestion of cellulose with a high‐purity recombinant cellulase, designated as E‐CELBA. After cellulase treatment, the S4B staining pattern of *trm4* mucilage changed from a disordered and tuft‐like ray structure to an ordered and diffuse ray structure. However, the ray lengths were still smaller than in WT (Fig. S5). RR staining also displayed the absence of dentate mucilage structure in *trm4* after E‐CELBA treatment (Fig. S5). E‐CELBA treatment of *trm4* mucilage cellulose microfibrils made the disordered cellulose deposition more diffuse, probably indicating that the deposition of unordered mucilage cellulose microfibrils is the reason for the *trm4* mucilage phenotype. It is therefore likely that TRM4 is involved in the alignment of mucilage cellulose microfibrils.

To compare the cellulose properties of *trm4* with other mutants exhibiting a compact mucilage phenotype, such as *csla2*,* ixr1‐2* and *muci10* (Fig. S6; Yu *et al*., 2014; Griffiths *et al*., 2015; Voiniciuc *et al*., 2015b), seeds were treated with an enzyme (E‐CELTR) that can hydrolyze β‐1,4‐glucan linkages in cellulose, as well as several hemicelluloses (Zietsman *et al*., 2015). RR staining of the buffer‐treated seeds displayed a compact mucilage capsule phenotype in all mutants (Fig. S7). The E‐CELTR treatment removed some, but not all, of the RR‐stained mucilage surrounding *trm4‐1* and *trm4‐2* seeds (Fig. S7). WT mucilage exhibited a thicker mucilage halo attached to the seed surface compared with *trm4* mutants after digestion (Fig. S7a–f), indicating that *trm4* mucilage cellulose is more accessible to cellulase treatment than that of WT. In contrast, almost no mucilage was detectable around E‐CELTR‐treated *csla2*,* ixr1‐2* and *muci10‐1* seeds (Fig. S7). This implies that *trm4* mucilage is more resistant than the mucilage of these mutants (which have distinct mucilage architectures) to cellulase treatment.

### Pectin spatial distribution is altered in *trm4* seeds

CCRC‐M30, a monoclonal antibody that binds *A. thaliana* mucilage (Pattathil *et al*., 2010), was used to determine the precise distribution of pectic polymers around seeds counterstained with S4B. CCRC‐M30 signals were primarily detected between the outer edges of cellulosic rays in WT and *trm4* seeds (Fig. S8). However, in *trm4* mucilage, the distribution of CCRC‐M30 labeling was reduced, forming a dentate pattern, which was also observed with RR staining (Figs 2, S8).

### Morphological observation of SCE cells

The SCE cells of *A. thaliana* mature seeds are hexagonal in shape outlined by secondary radial walls with a volcano‐shaped columella (Western *et al*., 2000). SEM was employed to observe the surface morphology of *trm4‐1* and WT dry seeds at maturity. No alterations were observed in the size of SCE cells, their radial walls or columellae (Fig. S9).

### The *trm4* mutants have a denser mucilage capsule independent of calcium‐mediated expansion

Driven by the previous analysis, we assumed that *trm4* displays a denser adherent mucilage layer because of the reduction in the adherent mucilage area and no altered chemical composition. To test this hypothesis, dextrans labeled with FITC were used to analyze mucilage porosity (Willats *et al*., 2001; Voiniciuc *et al*., 2015b). The 20‐kDa FITC‐dextran molecules reached the seed surface of all mutants and WT (Fig. S10). By contrast, 70‐kDa FITC‐dextran molecules were preferentially excluded from the *trm4* and *muci10* mucilage capsules, relative to WT (Fig. S10). This suggests that *trm4* seeds have denser adherent mucilage capsules, similar to the previously described *muci10‐1* mutant (Voiniciuc *et al*., 2015b).

Unesterified regions of HG polymers can be cross‐linked by calcium bridges to promote pectin gel formation. To determine whether the *trm4* compact mucilage phenotype resulted from the changes in calcium cross‐links, we analyzed the RR staining of seeds treated with the cation chelator EDTA or with CaCl_2_. The removal of cations by EDTA was not sufficient to expand the adherent mucilage capsule of *trm4* seeds to the WT level (Fig. S10). Calcium‐treated seeds of *trm4* also showed more compact mucilage compared with WT (Fig. S10), but mucilage capsules were still released from the SCE cells. Therefore, *trm4* mucilage was more compact than WT regardless of the presence of calcium, indicating that the denser mucilage capsule did not result from increased HG cross‐links.

### TRM4 proteins co‐localize with microtubules and are sensitive to oryzalin

**Figure 6:**
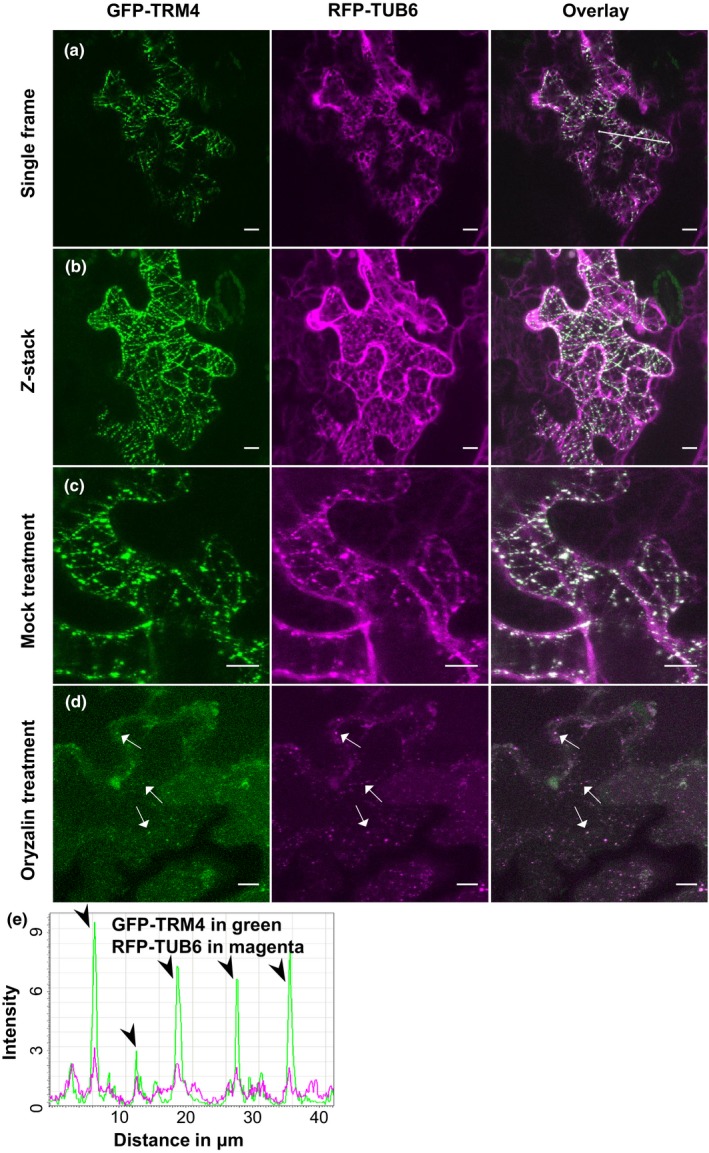
TONNEAU1 (TON1) recruiting motif 4 (TRM4) proteins are co‐localized with microtubules (MT) and sensitive to oryzalin treatment. The epidermal cells of tobacco leaves expressing GFP‐TRM4 and RFP‐TUB6: (a) single frame; (b) maximum projection of a *Z*‐stack; (c) maximum projection of a *Z*‐stack of mock treatment; (d) maximum projection of a *Z*‐stack of oryzalin treatment. The arrows show the dispersed but co‐localized puncta. (e) Intensity plot analysis of GFP‐TRM4 and RFP‐TUB6 from transect (white line) in (a). The arrowheads indicate that GFP‐TRM4 proteins coincide with RFP‐RUB6 proteins. Bars, 10 μm.

As several TRM proteins have been characterized as playing important roles in the maintenance of microtubule organization (Spinner *et al*., 2013), we hypothesized that TRM4 might function as a microtubule‐associated protein. To elucidate this role, the subcellular location of TRM4 was tested using a GFP‐tagged TRM4 (GFP‐TRM4) construct co‐transfected with the microtubule marker RFP‐TUB6 in epidermal cells of tobacco leaves. The GFP‐TRM4 proteins displayed striated patterns and were overlapped with the RFP‐TUB6 proteins (Fig. 6). Intensity plot analysis also showed that GFP‐TRM4 and RFP‐TUB6 proteins were co‐localized (Fig. 6). In addition, the subcellular localization of a TRM4 fragment showed that the central part of the TRM4 protein, TRM4F2 (341–670 amino acids of TRM4), labeled cortical microtubule arrays (Fig. S11). This result is consistent with the observation that a similar TRM1 fragment (342–586 amino acids of TRM1) plays a vital role in microtubule binding (Drevensek *et al*., 2012). Furthermore, if treated with the microtubule inhibitor oryzalin (Morejohn *et al*., 1987), resulting in severe disruption of microtubules, the defective microtubule organization led to the dispersal of GFP‐TRM4 proteins out of the linear arrays, as observed in the mock‐treated cells. However, GFP‐TRM4 and RFP‐TUB6 proteins were still co‐localized after oryzalin treatment (Fig. 6c,d).

### Implications for TRM4 involvement in the maintenance of microtubule organization

TRM proteins serve as connectors between TON1 proteins and microtubules (Drevensek *et al*., 2012). In order to characterize a potential interaction between TRM4 and microtubules, we crossed a *trm4‐1* plant with a RFP‐TUB6 plant. Multiple independent homozygous *trm4‐1* lines with RFP‐TUB6 signals were obtained and showed the identical compact mucilage capsule phenotype observed in the *trm4* mutant plant (Fig. S12).

**Figure 7:**
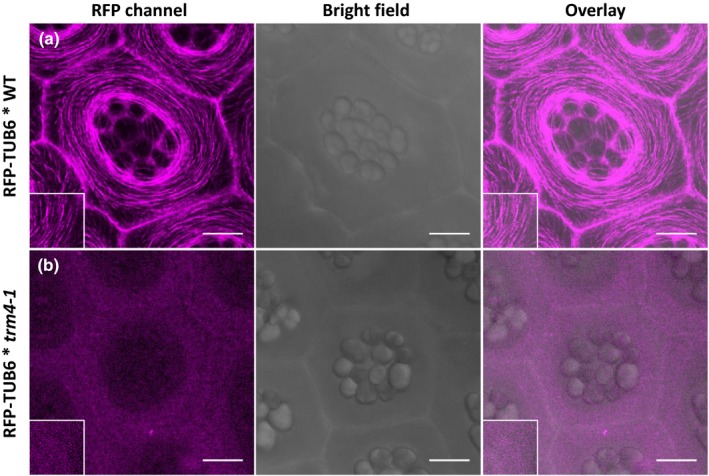
Microtubule distribution is disordered in seed coat epidermal (SCE) cells in *trm4‐1*. Representative results of RFP‐TUB6 distribution in the epidermal cells of the *Arabidopsis thaliana* seed coat in (a) wild‐type (WT) and (b) *trm4‐1* background at the stage of 7 d post‐anthesis (DPA) in the maximum projection of a *Z*‐stack view. The magnified insets are *c*. 10 μm in height and width. RFP, red fluorescent protein. Bars, 10 μm.

In the inner and outer faces of hypocotyl epidermal cells of zone 1 (Crowell *et al*., 2011), the microtubule orientations were not changed in the *trm4‐1* mutant background. Most microtubule arrays were perpendicular to the axis of the growing direction in both WT and *trm4‐1* backgrounds in the inner face of the hypocotyl (Fig. S13). However, the RFP‐TUB6 distribution in a *trm4‐1* background was disordered in the epidermal cells of seed coats. In WT SCE cells at the 7‐DPA stage, RFP‐TUB6 proteins were clearly observed and displayed in linear arrays around the cytoplasmic column (Figs 7a, S14). However, in all *trm4‐1* lines examined, the RFP‐TUB6 signals were faint and disordered (Figs. 7b, S14). The impaired RFP‐TUB6 distribution in the epidermal cells of *trm4* seed coats supports a vital role for TRM4 in the maintenance of microtubule organization.

### TRM4 proteins interact with CESA proteins

**Figure 8:**
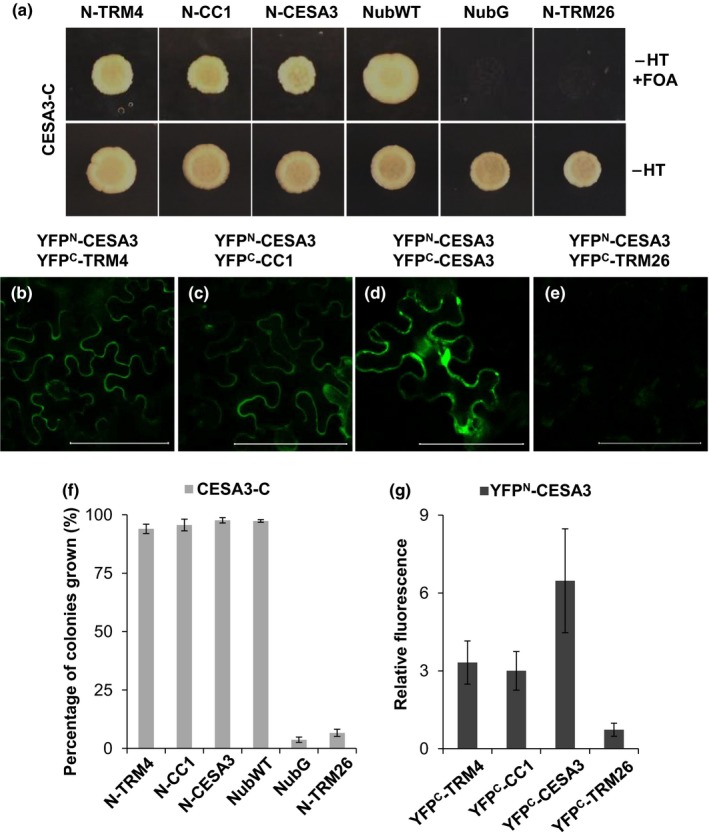
Interaction between TONNEAU1 (TON1) recruiting motif 4 (TRM4) and CESA3. (a) Yeast two‐hybrid analyses detect the interaction between TRM4 and CESA3. Yeast cells were selected on synthetic complete medium minus histidine and tryptophan (SC‐HT) with 5‐fluoroorotic acid (5‐FOA) for 5 d. N‐CC1, N‐CESA3 and NubWT are positive controls. NubG and N‐TRM26 are negative controls. (b–e) Bimolecular fluorescence complementation (BiFC) analyses confirm the interaction between TRM4 and CESA3 in the epidermal cells of tobacco leaves. (b) YFP^N^‐CESA3 with YFP^C^‐TRM4. (c, d) Positive controls YFP^C^‐CC1 and YFP^C^‐CESA3. (e) Negative control YFP^C^‐TRM26. Bars, 100 μm. (f) Percentage of colonies grown after 3 d at 30°C on SC‐HT medium with 5‐FOA. (g) Relative fluorescence yield from BiFC experiments. Values represent means ± SD of three independent replicates.

As CESA3/5 and TRM4 proteins coincide with microtubules, and the mutants all display affected mucilage extrusion (Sullivan *et al*., 2011; Griffiths *et al*., 2015), we further examined whether TRM4 can interact with CESA3 using the yeast split‐ubiquitin two‐hybrid approach. CESA3 proteins were fused to the C‐terminal part of ubiquitin (Cub) and the URA3 reporter protein for use as bait (CESA3‐C). TRM4, COMPANION OF CELLULOSE SYNTHASE 1 (CC1; Endler *et al*., 2015), CESA3 and TRM26 were fused to the C‐terminus of the mutated N‐terminal part of ubiquitin (NubG) for use as prey (N‐Prey). The coexpression of CESA3‐C and N‐TRM4 in yeast cells conferred a 5‐FOA resistance, revealing an interaction between CESA3 and TRM4 (Fig. 8a,f). However, no interaction was detected between CESA3 and TRM26, a member of the TRM family. CC1, CESA3 and NubWT were included as positive controls and TRM26 and NubG served as negative controls (Fig. 8a,f).

To confirm the interactions *in planta*, BiFC analysis was implemented. The coexpression of YFP^N^‐CESA3 (the N‐terminal half of YFP fused to the N‐terminus of CESA3) and YFP^C^‐TRM4 (the C‐terminal half of YFP fused to the N‐terminus of TRM4) in tobacco leaves led to the detection of YFP fluorescence, verifying the interaction between CESA3 and TRM4 (Fig. 8b,g). Similar results were obtained in the pairs of interacting proteins (YFP^N^‐CESA3 and YFP^C^‐CC1; YFP^N^‐CESA3 and YFP^C^‐CESA3) used as positive controls (Fig. 8c,d,g). No YFP fluorescence was detected in the co‐expression of YFP^N^‐CESA3 and YFP^C^‐TRM26, which served as negative controls (Fig. 8e,g).

## Discussion

Mucilage cellulose is deposited around the cytoplasmic column circumferentially by CESA complexes guided by microtubules (Griffiths *et al*., 2015). Our data reveal that TRM4 is an important player in the maintenance of microtubule organization in SCE cells and in sustaining the correct cellulose deposition in mucilage. *In vitro* assays confirm that TRM4 proteins can co‐align with microtubules and interact with CESA3. These results indicate a role for TRM4 in the organization of microtubules, which is required for cellulose deposition and mucilage organization.

### TRM4 influences the organization of cellulose microfibrils in SCE cells

Compared with WT, the shortened cellulosic rays are disordered and the diffuse labeling between and on top of the rays is not evident in *trm4* (Fig. 3). This implies that cellulose microfibril deposition is disordered in *trm4*. CBM3a immunolabeling displays mushroom cap‐like structures on the top of rays in *trm4* mucilage, indicating that the crystalline cellulose microfibrils are restrained (Fig. 4). The results of S4B staining, CBM3 labeling and birefringence analysis (Figs 3, 4, 5) indicate that TRM4 influences the deposition and organization of mucilage cellulose microfibrils. This role is further supported by the interaction between CESA3 and TRM4 proteins at the molecular level (Fig. 8). In addition, the treatment of *trm4* seeds with high‐purity cellulose renders the disordered tuft‐like rays more diffuse (Fig. S5). It is probable that, in *trm4*, the mucilage cellulose microfibrils are misaligned and restrained during cellulose biosynthesis, resulting in short and disordered rays without the diffuse labeling between and on top of the cellulosic rays. The cellulase can partially digest the misaligned cellulose microfibrils to render a diffuse cellulose architecture. Together, these results indicate that TRM4 plays an important role in the maintenance of cellulose microfibril alignment and deposition in mucilage synthesis.

Although TRM4 influences cellulosic structure in extruded mucilage, we detected no defects in SCE cell morphology in the *trm4‐1* mutant (Fig. S9). By contrast, overexpression of *TRM1* and *TRM2*, which are clustered in the same group as *TRM4* (Fig. S2), can increase the longitudinal cell elongation in different plant organs (Lee *et al*., 2006; Drevensek *et al*., 2012). However, unlike *TRM1* and *TRM2*, overexpression of *TRM4* in *trm4‐1* does not result in dramatic changes in vegetative tissues (data not shown), or at the seed surface (Fig. S9). Hence, TRM4 appears to specifically affect the cellulose deposition in mucilage pockets, rather than SCE cell elongation. It has been shown that TRM1 is essential for microtubule organization and is able to recruit TON1 and FASS/TON2 to microtubules via its C‐terminal M2 and M3 motifs, respectively (Drevensek *et al*., 2012; Spinner *et al*., 2013). TRM4 has also been identified in the TTP complex, interacting with the TON1b isoform and FASS/TON2 (Spinner *et al*., 2013). Therefore, the aberrant cellulose distribution around *trm4* seeds probably results from disorganized microtubule arrays in SCE cells during mucilage production.

### Mucilage cellulose deposition is vital to sustain mucilage structure and extrusion

A number of studies of mutants with cellulose content defects have demonstrated that cellulose synthesis plays an important role in mucilage adherence in seeds and mucilage extrusion (Harpaz‐Saad *et al*., 2011; Mendu *et al*., 2011; Sullivan *et al*., 2011; Ben‐Tov *et al*., 2015; Griffiths *et al*., 2015). For example, the *ixr1‐2* mutation in *CESA3* results in seeds with compact mucilage capsules, short cellulosic rays and a mild reduction in cellulose content (Griffiths *et al*., 2015). Mutations in *CSLA2* or *MUCI10*, which are essential for mucilage galactoglucomannan synthesis, result in a compact mucilage capsule and slightly lower mucilage cellulose and hemicellulose content (Yu *et al*., 2014; Voiniciuc *et al*., 2015b). Similarly, loss‐of‐function mutations in *TRM4* lead to a compact mucilage capsule, short cellulosic rays and disordered cellulose deposition in SCE cells. The microfibril misalignment in *trm4* alters the pectin distribution, but does not affect the abundance of crystalline cellulose, hemicelluloses and pectin in mucilage (Figs 3o, S8; Table S2). Cellulase treatments have been used to characterize changes in cellulose properties. E‐CELBA is a high‐purity recombinant cellulase that can degrade crystalline cellulose, whereas E‐CELTR can hydrolyze cellulose and hemicelluloses (Zietsman *et al*., 2015). E‐CELBA can specifically hydrolyze cellulosic rays to make them more diffuse in appearance. Interestingly, E‐CELBA‐treated WT seeds phenocopy the cellulose architecture of *sos5* seeds, which lack clearly defined rays (Fig. S5; Griffiths *et al*., 2014). Relative to WT, the *trm4* mucilage is more susceptible to E‐CELTR digestion. A possible explanation is that the misaligned microfibrils in *trm4* mucilage are more accessible to the cellulase enzymes. In contrast with the *trm4* alleles, the *ixr1‐2*,* csla2* and *muci10* mutant seeds display almost no adherent mucilage after E‐CELTR treatment (Fig. S7). Therefore, the mucilage is easier to remove from mutant seeds that are already deficient in the content of at least one mucilage polysaccharide. Unlike *trm4*, the loss of hemicelluloses in mucilage (as in *csla2* and *muci10*) results in an absolute reduction in the content of crystalline cellulose. Hemicelluloses are typically involved in cross‐linking cellulose microfibrils, and can thus influence cellulose synthesis and its final architecture. In support of this hypothesis, glucomannan with variable substitutions forms cross‐links with cellulose microfibrils *in vitro*, which affects the crystallinity and ultrastructure of cellulose (Whitney *et al*., 1998). Although tight association between mucilage organization and cellulose content in mucilage has been detected previously, our study suggests that irregular deposition of cellulose in *trm4* mutants can also have a profound impact on mucilage architecture.

### TRM4 facilitates cellulose alignment by the maintenance of microtubule organization

There is mounting evidence to support the hypothesis that cellulose microfibrils are aligned with underlying cortical microtubules perpendicular to the direction of the maximal expansion rate in the growing cell wall (Green, 1962; Ledbetter & Porter, 1963; Baskin *et al*., 2004; Bringmann *et al*., 2012a). Microtubule inhibitor treatment of *Arabidopsis* root cells shows that disorganized cortical microtubules increase the rate of tangential expansion and reduce the uniformity of cellulose microfibrils (Baskin *et al*., 2004). The disruption of *FRAGILE FIBER 2* (*FRA2*), which encodes a katanin‐like protein regulating microtubule organization, leads to the aberrant deposition of cellulose microfibrils in the secondary cell wall and defects in cell elongation (Burk & Ye, 2002).

In this study, we found that the distribution of the microtubule marker RFP‐TUB6 is disorganized in the epidermal cells of *trm4* seed coats (Figs 7, S14). This demonstrates that TRM4 is essential for the maintenance of the organization of microtubules in SCE cells. In addition, GFP‐TRM4 proteins coincide with microtubule arrays and are sensitive to oryzalin treatment (Fig. 6), implying that TRM4 functionally resembles TRM1. Presumably, TRMs serve as an assembly or targeting component of the TTP complex, which is the main regulator of microtubule organization (Schaefer *et al*., 2017). On the one hand, TRMs can interact directly with microtubules through the microtubule binding domain (Drevensek *et al*., 2012). On the other, TRMs can recruit TON1 and FASS, two essential components in the maintenance of microtubule organization, to the microtubule via M2 and M3 motifs, respectively. (Drevensek *et al*., 2012; Spinner *et al*., 2013; Schaefer *et al*., 2017).

Our identification and functional characterization of TRM4 expands the model proposed for the deposition of cellulose in seed mucilage. In SCE cells at the 7‐DPA stage, the CSCs track along the microtubules, moving around the cytoplasmic column circumferentially (Griffiths *et al*., 2015). CELLULOSE SYNTHASE INTERACTING1 (CSI1), which functions as a molecular bridge between microtubules and CSCs, may facilitate the guidance of the CSCs along the microtubules in this context because of its high expression in SCE cells (Bringmann *et al*., 2012b; Li *et al*., 2012; Griffiths & North, 2017). However, the mechanism that maintains microtubule array dynamics and organization in SCE cells is unclear. We have discovered that TRM4 is required for the maintenance of microtubule organization in this cell type at the peak of mucilage polysaccharide production, and to sustain the orientation of cellulose deposition via direct interactions with CESA3. CESA3, together with CESA5, is an essential component of the CSCs that synthesize cellulose in SCE cells (Griffiths *et al*., 2015; Griffiths & North, 2017). During cellulose synthesis, one possible scenario may be that TRM4 maintains microtubule organization by the TTP complex and recruits CSCs to the microtubule through the specific interaction motif. Meanwhile, CSI1 sustains CSC activity along the microtubule and facilitates a connection between CESA and the microtubule. To support this hypothesis, further investigations are required to determine the roles of these two microtubule‐associated proteins in mucilage cellulose synthesis. In hydrated seeds, the cellulose microfibrils and tightly associated pectins in the mucilage pockets are released as hydrophilic capsules, which are, at least partially, anchored and shaped by cellulosic rays. Thus, TRM4 plays an important role in the orientation of cellulose deposition and ray length in SCE cells during mucilage synthesis, which consequently affects mucilage architecture. As TRM4 proteins have seven motifs and can recruit other proteins to microtubules through their motifs (Drevensek *et al*., 2012; Spinner *et al*., 2013), the TRM4 protein may have additional functions. Therefore, additional studies are necessary to fully elucidate the interacting partners of TRM4 and its biological roles.

## Author contributions

BY, CV and BU designed the research. LF performed elastic‐net co‐expression analysis. SD performed SEM. CV performed RR staining, mucilage area quantification, FITC‐dextran staining and monosaccharide analysis. BY performed the other experiments and wrote the article. CV, HK and BU supervised the project and revised the manuscript. All authors discussed the results and approved the final manuscript.

## Supporting information

Please note: Wiley Blackwell are not responsible for the content or functionality of any Supporting Information supplied by the authors. Any queries (other than missing material) should be directed to the *New Phytologist* Central Office.

**Fig. S1** Expression pattern of *TRM4*.
**Fig. S2** Phylogenetic tree of the *TRM* family in *Arabidopsis thaliana*,* Solanum lycopersicum* and *Oryza sativa*.
**Fig. S3** Mutant identification of *TRM4* and its paralog *TRM3*.
**Fig. S4** Complementation of *TRM4* can rescue mucilage defects in *trm4‐3* and *trm4‐3 trm3‐1*.
**Fig. S5** Mild cellulase digestion makes *trm4‐1* mucilage cellulose more diffuse.
**Fig. S6** Ruthenium red (RR) staining of wild‐type (WT), *trm4‐1*,* trm4‐2*,* csla2‐3*,* muci10‐1* and *ixr1‐2* seed mucilage.
**Fig. S7** *trm4* shows more resistance than *csla2*,* ixr1‐2* and *muci10* on cellulase digestion.
**Fig. S8** Immunolabeling of mucilage pectin in adherent mucilage.
**Fig. S9** Micrograph of seed coat epidermal cells by scanning electron microscopy.
**Fig. S10** *trm4* mutants have a denser mucilage capsule independent of calcium‐mediated expansion.
**Fig. S11** The central part of TRM4 is essential for microtubule subcellular localization.
**Fig. S12** *trm4‐1* plants carrying RFP‐TUB6 show a compact mucilage phenotype.
**Fig. S13** Microtubule organization in the inner and outer faces of epidermal cells in the hypocotyl.
**Fig. S14** Microtubule distribution in multiple seed coat epidermal (SCE) cells.
**Table S1** Primers used in this study.
**Table S2** Monosaccharide composition of non‐adherent mucilage and total mucilage.
