# Supplementary material for: TRM4 is essential for cellulose deposition in Arabidopsis seed mucilage by maintaining cortical microtubule organization and interacting with CESA3
Source: New Phytol. 2018 Sep 13;221(2):881–95. doi: 10.1111/nph.15442 (PMC6585848; doi:10.1111/nph.15442)
Supplement: Supplementary file 1 — Fig. S1 Expression pattern of TRM4. Fig. S2 Phylogenetic tree of the TRM family in Arabidopsis thaliana, Solanum lycopersicum and Oryza sativa. Fig. S3 Mutant identification of TRM4 and its paralog TRM3. Fig. S4 Complementation of TRM4 can rescue mucilage defects in trm4‐3 and trm4‐3 trm3‐1. Fig. S5 Mild cellulase digestion makes trm4‐1 mucilage cellulose more diffuse. Fig. S6 Ruthenium red (RR) staining of wild‐type (WT), trm4‐1, trm4‐2, csla2‐3, muci10‐1 and ixr1‐2 seed mucilage. Fig. S7 trm4 shows more resistance than csla2, ixr1‐2 and muci10 on cellulase digestion. Fig. S8 Immunolabeling of mucilage pectin in adherent mucilage. Fig. S9 Micrograph of seed coat epidermal cells by scanning electron microscopy. Fig. S10 trm4 mutants have a denser mucilage capsule independent of calcium‐mediated expansion. Fig. S11 The central part of TRM4 is essential for microtubule subcellular localization. Fig. S12 trm4‐1 plants carrying RFP‐TUB6 show a compact mucilage phenotype. Fig. S13 Microtubule organization in the inner and outer faces of epidermal cells in the hypocotyl. Fig. S14 Microtubule distribution in multiple seed coat epidermal (SCE) cells. Table S1 Primers used in this study. Table S2 Monosaccharide composition of non‐adherent mucilage and total mucilage. [file NPH-221-881-s001.pdf]

***New Phytologist* Supporting Information**

Article title: **TRM4 is essential for cellulose deposition in Arabidopsis seed mucilage by maintaining cortical microtubules organization and interacting with CESA3**

Authors: Bo Yang, Cătălin Voiniciuc, Lanbao Fu, Sabine Dieluweit, Holger Klose, Björn Usadel

Article acceptance date: 10 August 2018

The following Supporting Information is available for this article:

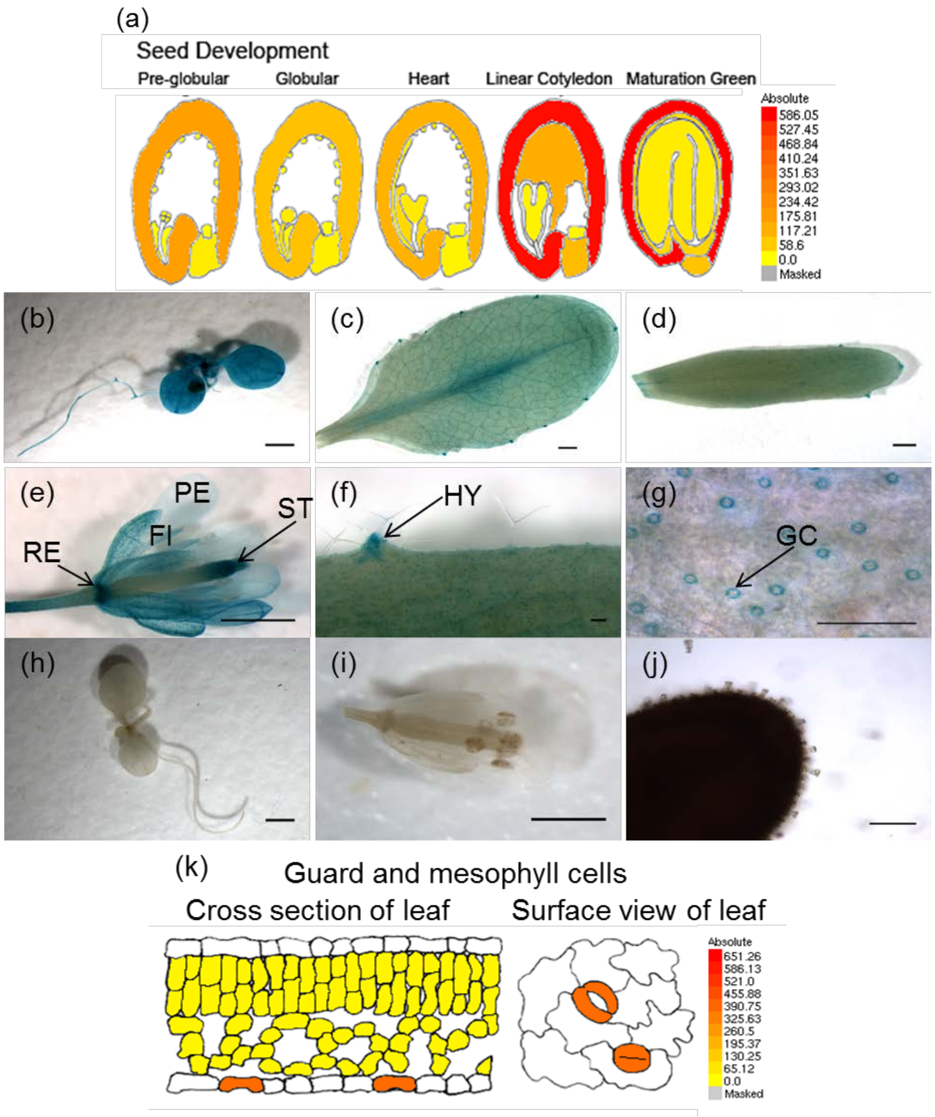

Fig. S1 Expression pattern of *TRM4*.

(a) *TRM4* transcript levels in the developing seed from the Arabidopsis eFP browser. (b-g) *pTRM4-GUS* staining. (b) Five days seedling. (c) Rosette leaf. (d) Cauline leaf. (e) Flower with GUS activity in receptacle (RE), stigma (ST), petals (PE) and filaments (FI). (f-g) Detailed images of cauline leaf showing GUS activity in hydathodes (HY) and guard cells (GC). (h-j) Negative control plant transformed with empty construct. (k) *TRM4* has strong expression in guard cells from visualized *Arabidopsis* eFP browser. Bars (b-e, h and i) 1000 μm; (f, g and j) 100 μm.

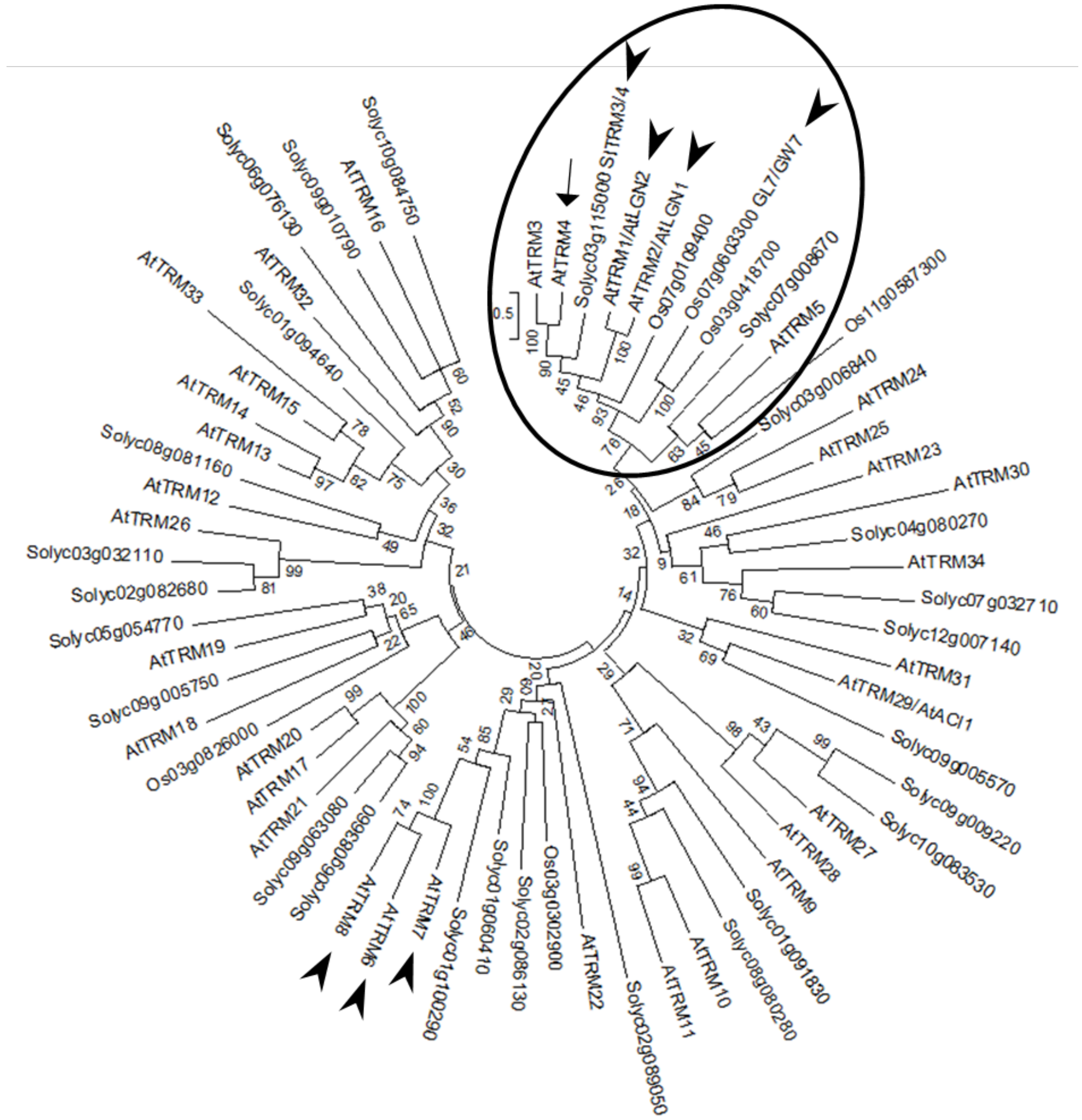

Fig. S2 Phylogenetic tree of *TRM* family in *A. thaliana* , *S. lycopersicum* and *O. sativa*.

The protein sequences are aligned by MUSCLE method. The tree is generated by MEGA6.0 software. The tree is drawn to scale, with branch lengths measured in the number of substitutions per site. Bootstrap values indicate the reliability of the nodes. Arrowheads indicate functional characterized *TRM* genes in the three species. Arrow points to *AtTRM4*.

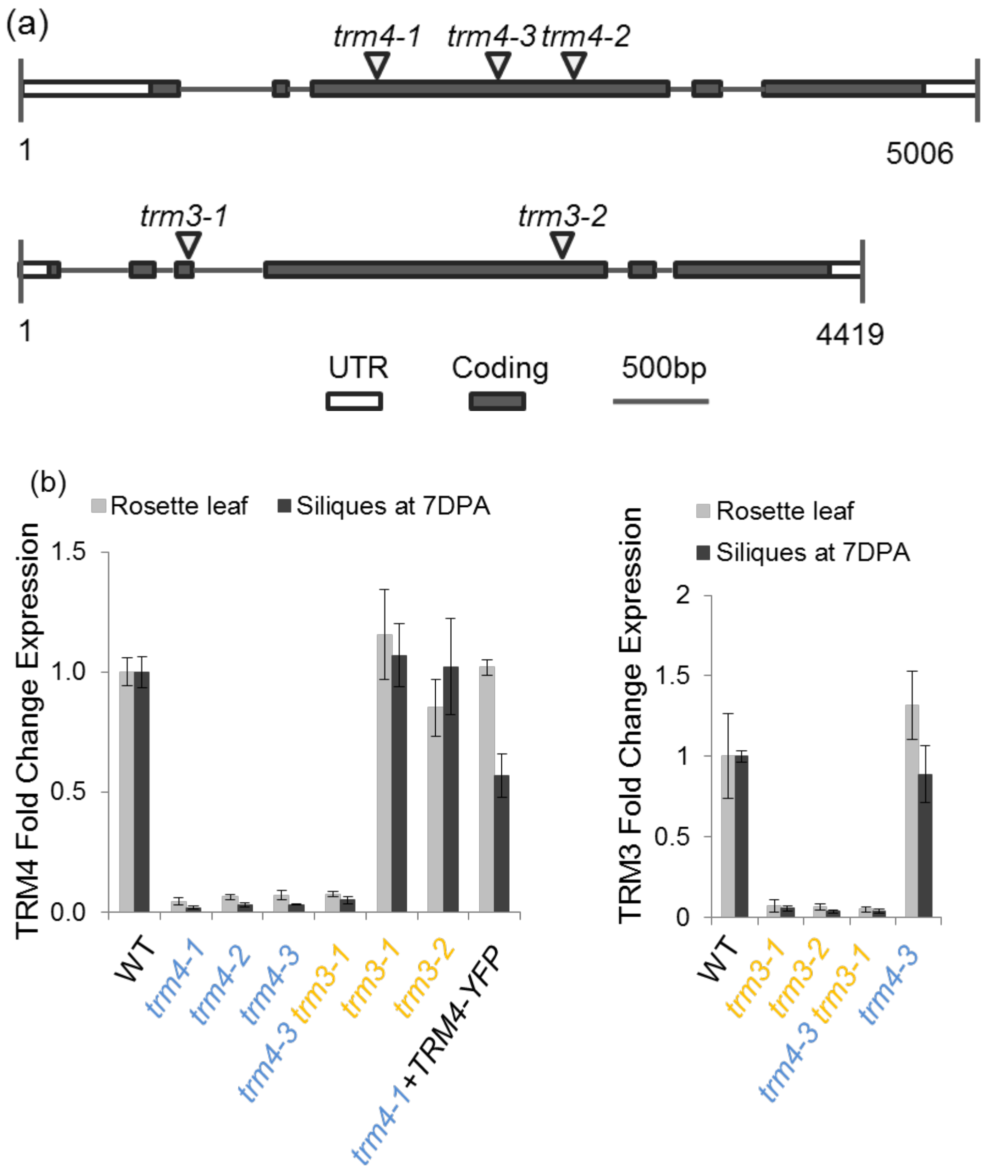

Fig. S3 Mutants identification of *TRM4* and its paralog *TRM3*.

(a) Schematic demonstration of gene structures of *TRM4* and *TRM3*. The triangles indicate the T-DNA insertion sites of mutants. (b) qRT-PCR analyses of gene expression in rosette leaves (five weeks old plants) and siliques at the 7DPA stage of WT, mutants and complementation lines with TRM4-YFP driven by *TRM4* promoter. Gene expression (normalized to *UBIQUITIN10*) relative to the first wild type in each set. Data show means ± SD of 3 biological replicates.

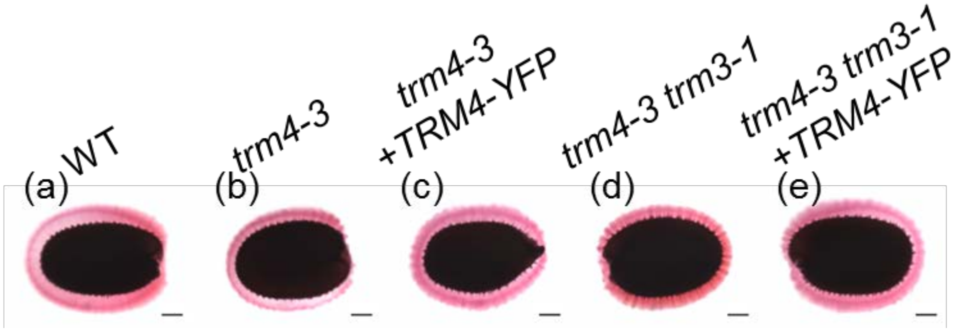

Fig. S4 Complementation of *TRM4* can rescue mucilage defects in *trm4-3* and *trm4-3 trm3-1*. The *TRM4-YFP* transgene was driven by *TRM4* promoter. (a-e) Ruthenium red stained seeds after hydration of WT, mutants and complemention lines driven by *TRM4* promoter, Bars = 100 μm.

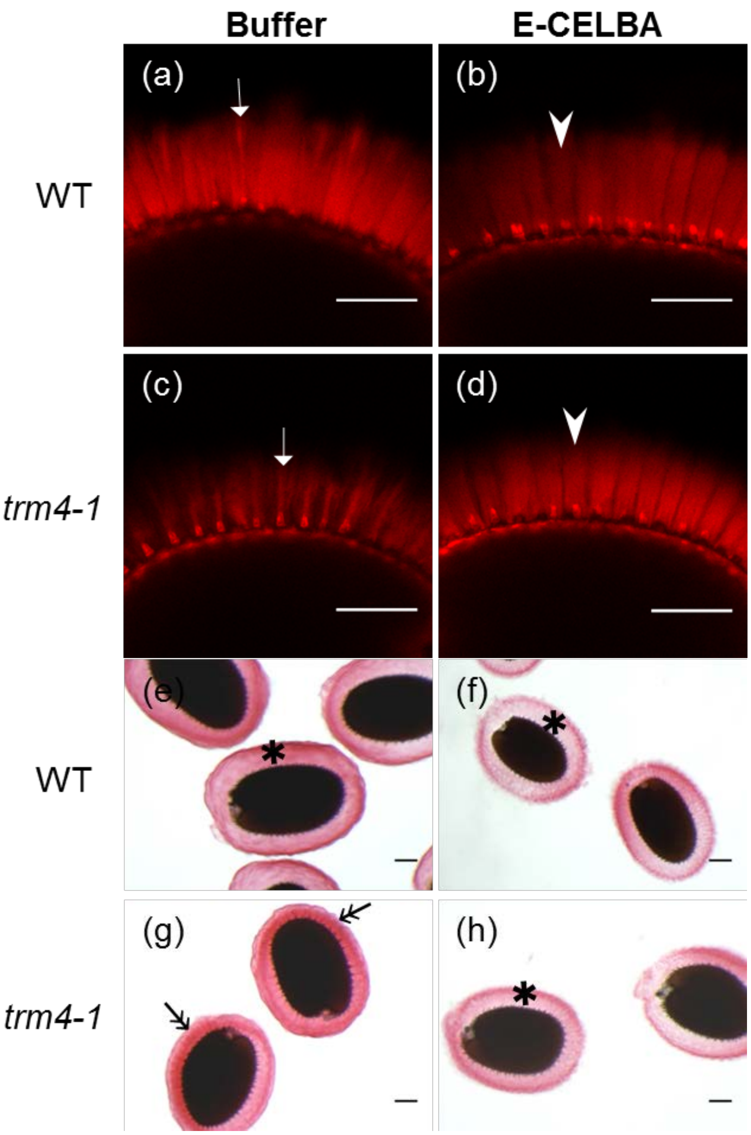

Fig. S5 Mild cellulase digestion makes *trm4-1* mucilage cellulose more diffused.

(a-d) S4B staining after 0.1 M phosphate buffer pH 6.0 treatment (a and c) or mild cellulase E-CELBA (endo-1,4-β-D-glucanase) (*B. amyloliquefaciens*) digestion for 90 min (b and d). (e-h) Ruthenium red staining after buffer treatment (e and g) or E-CELBA digestion (f and h). Arrows mark S4B stained rays. Arrowheads mark S4B stained diffused region. Double arrows mark ruthenium red stained dentate mucilage. Asterisks mark ruthenium red stained diffused region. Bars = 100 μm.

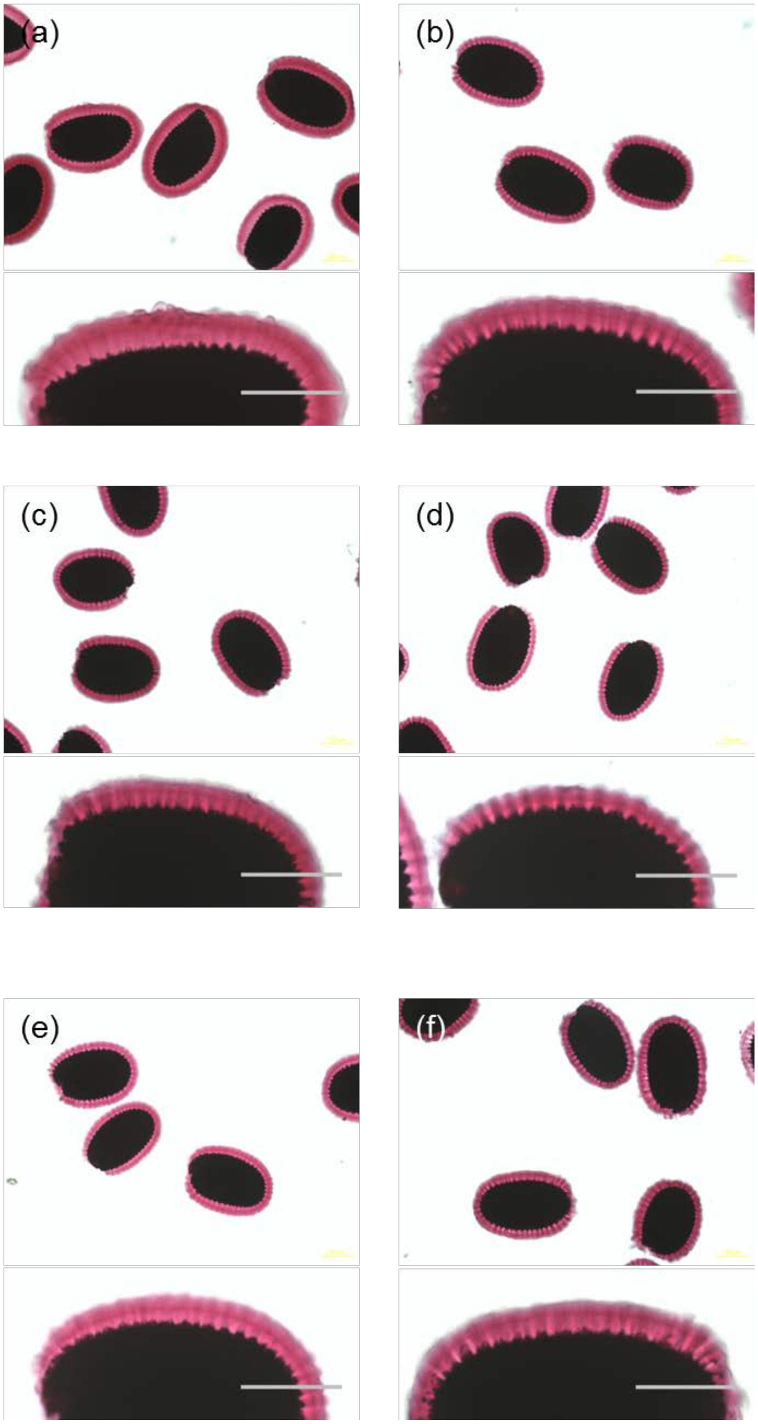

Fig. S6 Ruthenium red staining of WT, *trm4-1*, *trm4-2*, *csla2-3*, *muci10-1* and *ixr1-2* seed mucilage.

(a) WT. (b-f) *Trm4-1*, *trm4-2*, *csla2-3*, *muci10-1* and *ixr1-2* seeds showed compact mucilage capsule compared with WT after gently shaking in water. Bars = 200 μm.

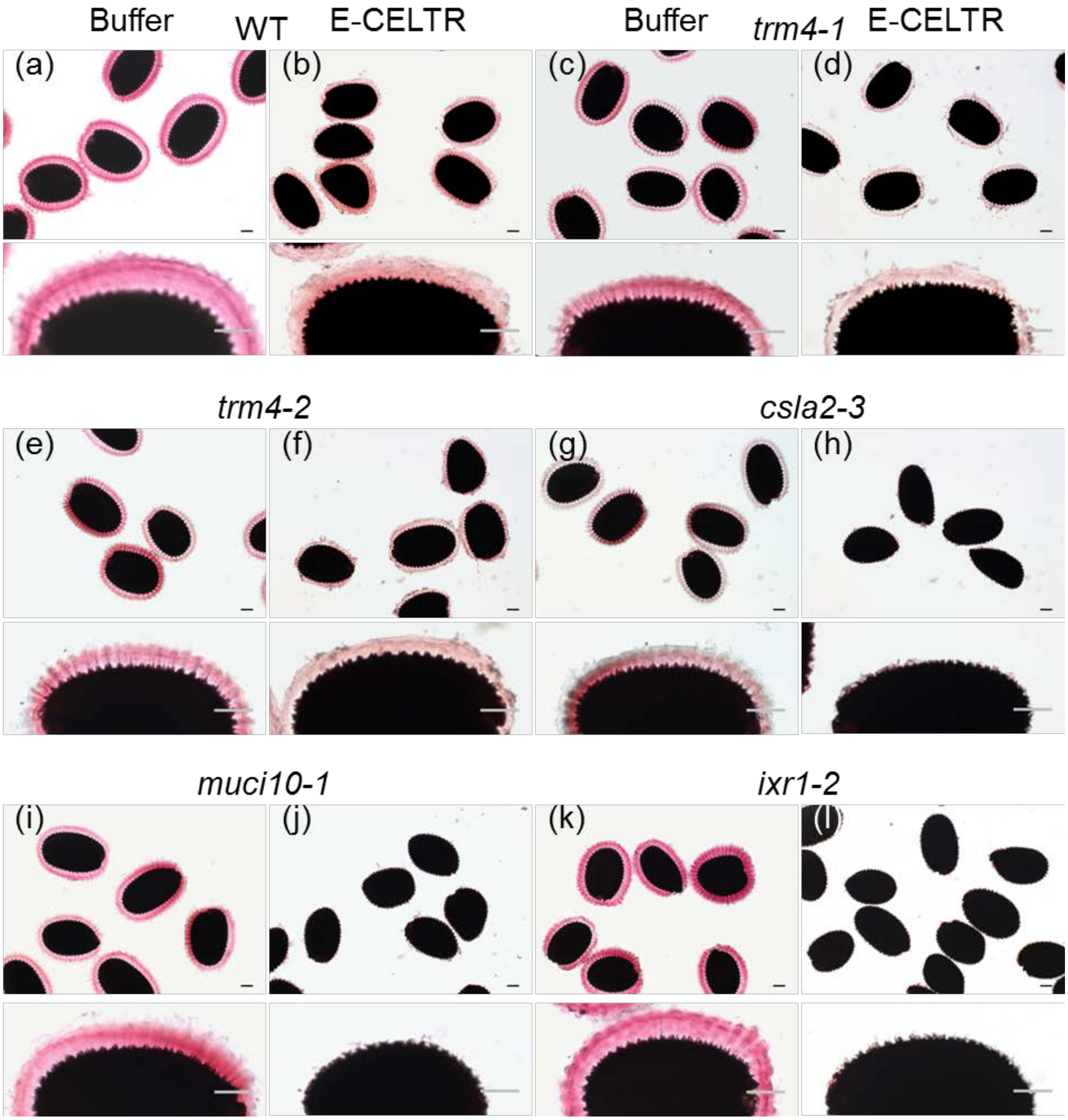

Fig. S7 *Trm4* shows more resistance on cellulase digestion than *csla2*, *ixr1-2* and *muci10*.

(a, c, e, g, i, k) Ruthenium red stained seeds after 0.1 M sodium acetate buffer pH 4.5 treatment for 90 min. (b, d, f, h, j, l) Ruthenium red stained seeds after E-CELTR (endo-1,4-β-D-glucanase) (*T. longibrachiatum*) treatment for 90 min. Bars = 100 μm.

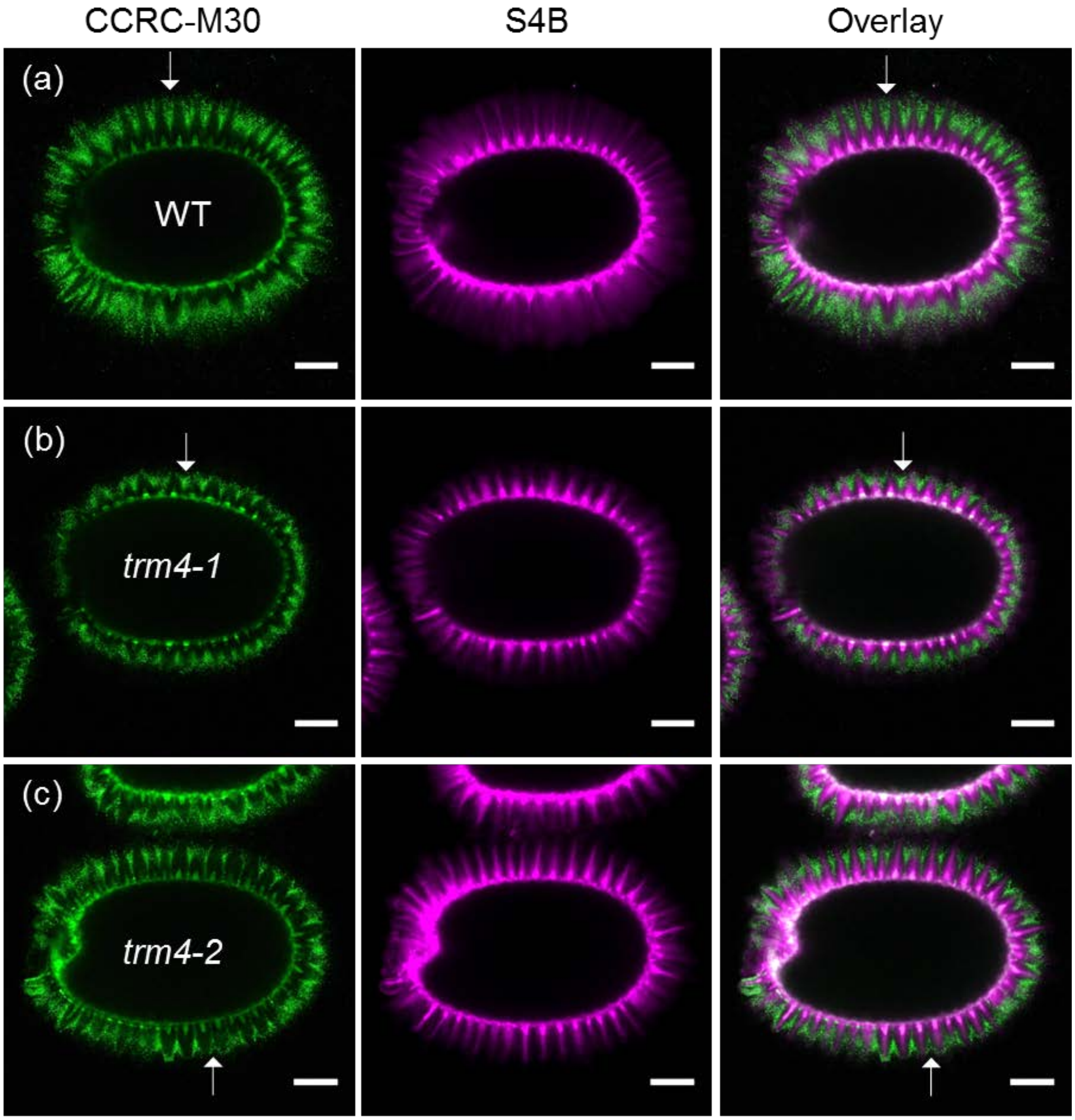

Fig. S8 Immunolabeling of mucilage pectin in adherent mucilage.

(a-c) Mucilage pectin was labeled with CCRC-M30 (green) and cellulose was labeled with S4B as counterstain (magenta) in (a) WT and (b-c) *trm4* seeds. The arrows indicate pectin labeling in the edge area between cellulosic rays. (b-c) In *trm4* seeds, mucilage distribution was reduced, forming a dentate pattern compared with the WT seeds. Bars = 100 µm.

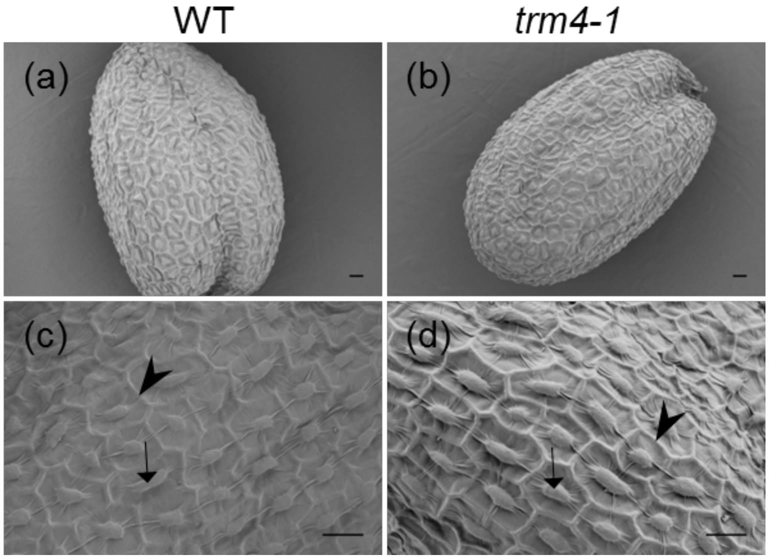

Fig. S9 Micrograph of seed coat epidermal cells by scanning electron microscope.

(a and c) WT seed surface. (b and d) *trm4-1* seed surface. Arrows indicate columella. Arrowheads mark radial wall. Bars = 20 μm.

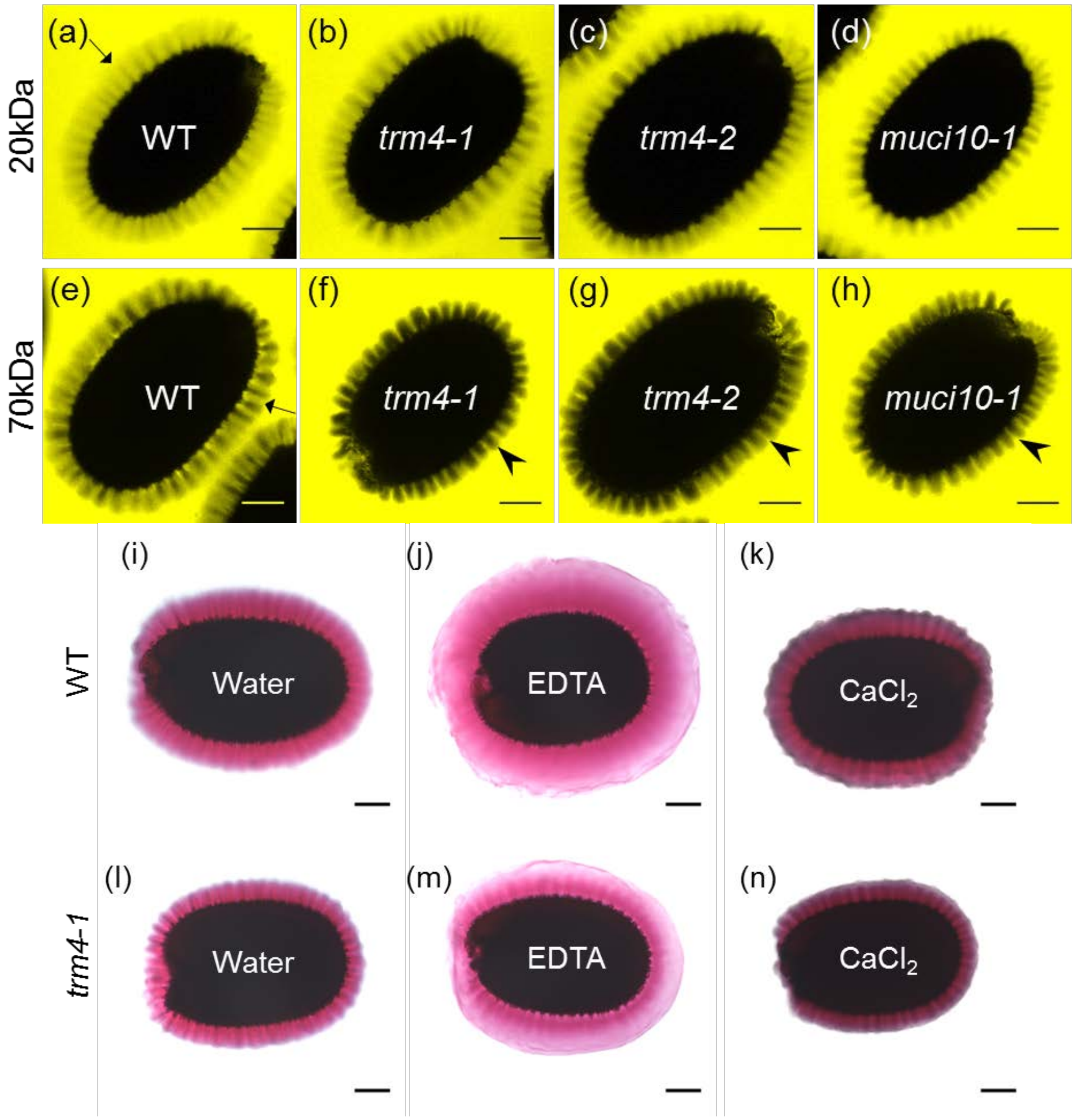

Figure S10. *Trm4* mutants have denser mucilage capsule independently of calcium-mediated expansion.

(a-d) 20-KDa FITC-dextran molecules (yellow) were excluded from the seed. (e-h) 70-KDa FITC-dextran molecules (yellow) were excluded from the seed, the rays (arrows) or the wide mucilage columns (arowheads). (i-n), Ruthenium red stained seeds after water, EDTA and $CaCl_2$ treatments. Bars = 100 µm.

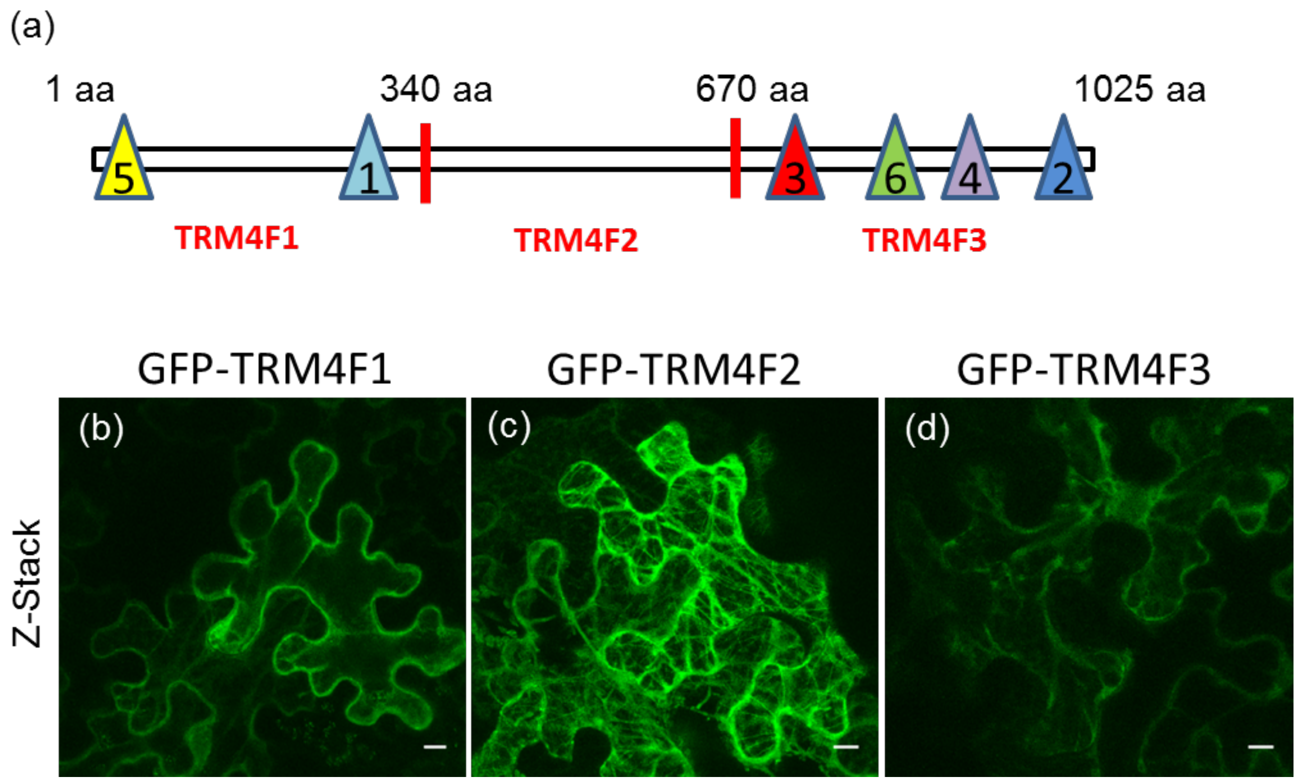

Fig. S11 The central part of TRM4 is essential for microtubule subcellular localization.

(a) The schematic diagram of TRM4 protein with six motifs as described (Drevensek *et al.*, 2012).

TRM4F1: the N-terminal of the TRM4 protein (the first 340 amino acids)

TRM4F2: the central part of the TRM4 protein (341 aa – 670 aa)

TRM4F3: the C-terminal of the TRM4 protein (671 aa – 1025 aa)

(b-d) The subcellular localization of truncated fragments of TRM4 in the epidermal cells of tobacco leaves. Bars = 10 µm.

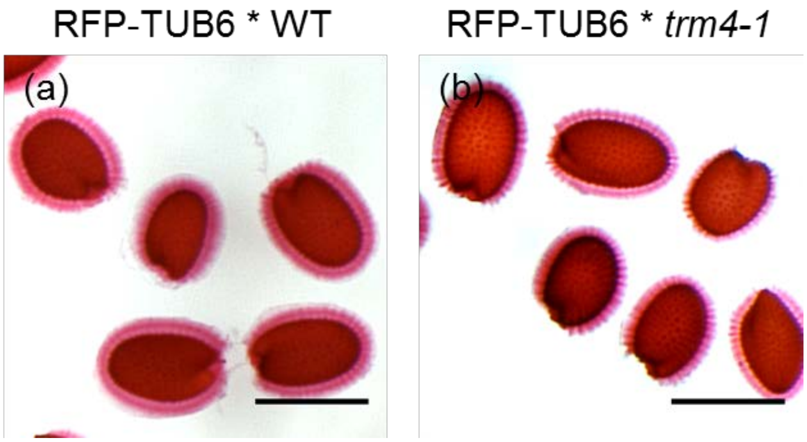

Fig. S12 *Trm4-1* plants carrying RFP-TUB6 show compact mucilage phenotype.

(a) Ruthenium red stained seeds of RFP-TUB6 in WT plant. (b) Ruthenium red stained seeds of RFP-TUB6 in homozygous *trm4-1* plant. Bars = 600 μm.

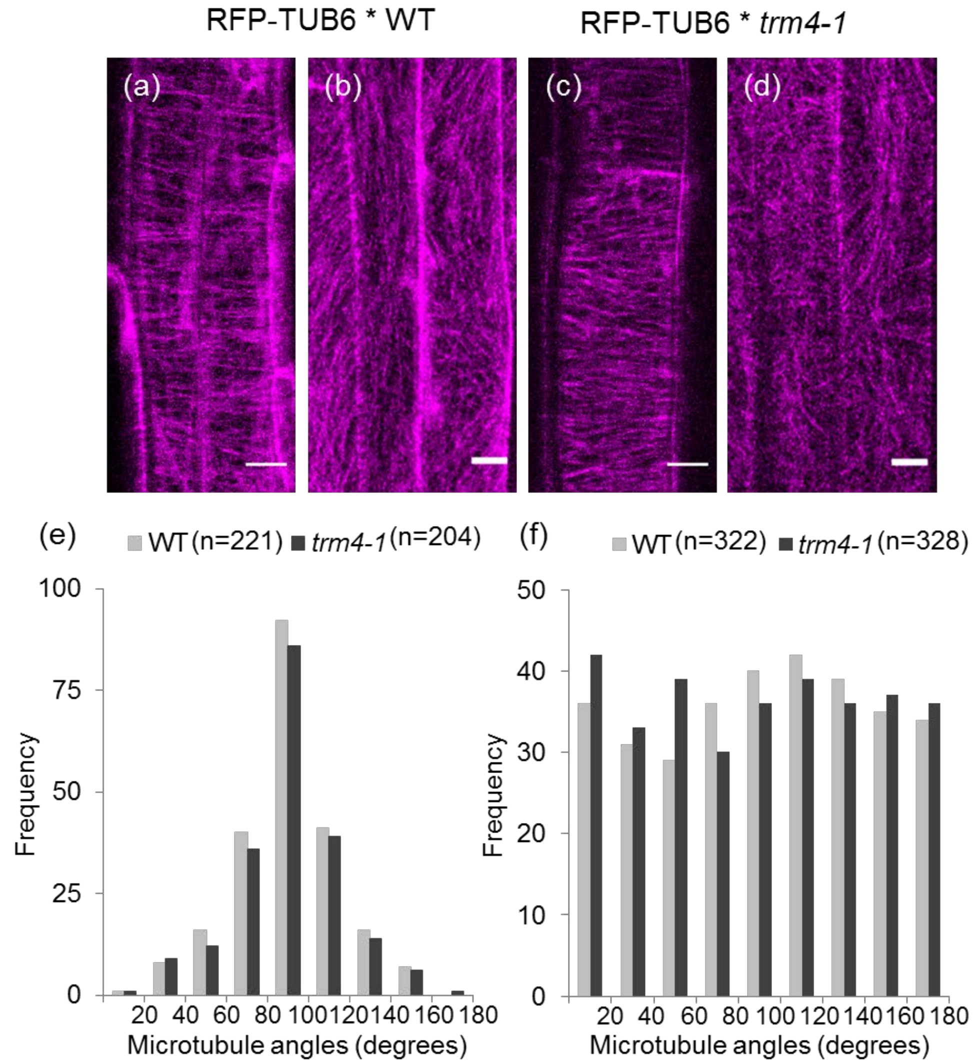

Fig. S13 Microtubule organization in the inner face and outer face of epidermal cells in hypocotyl.

(a-d) Maximum projected z-stacks demonstrating RFP-TUB6-labeled microtubules in inner face (a, c) and outer face (b, d) in hypocotyl zone 1 of WT plant (a, b) and homozygous *trm4-1* plant (c, d). Images are representative of n > 10 hypocotyls sampled. (e-f) The microtubule angle distribution in the inner face (e) and outer face (f) in hypocotyl cells . Angles were measured against the growth axis. Six cells from six seedlings were used for analysis. Bars = 10 μm.

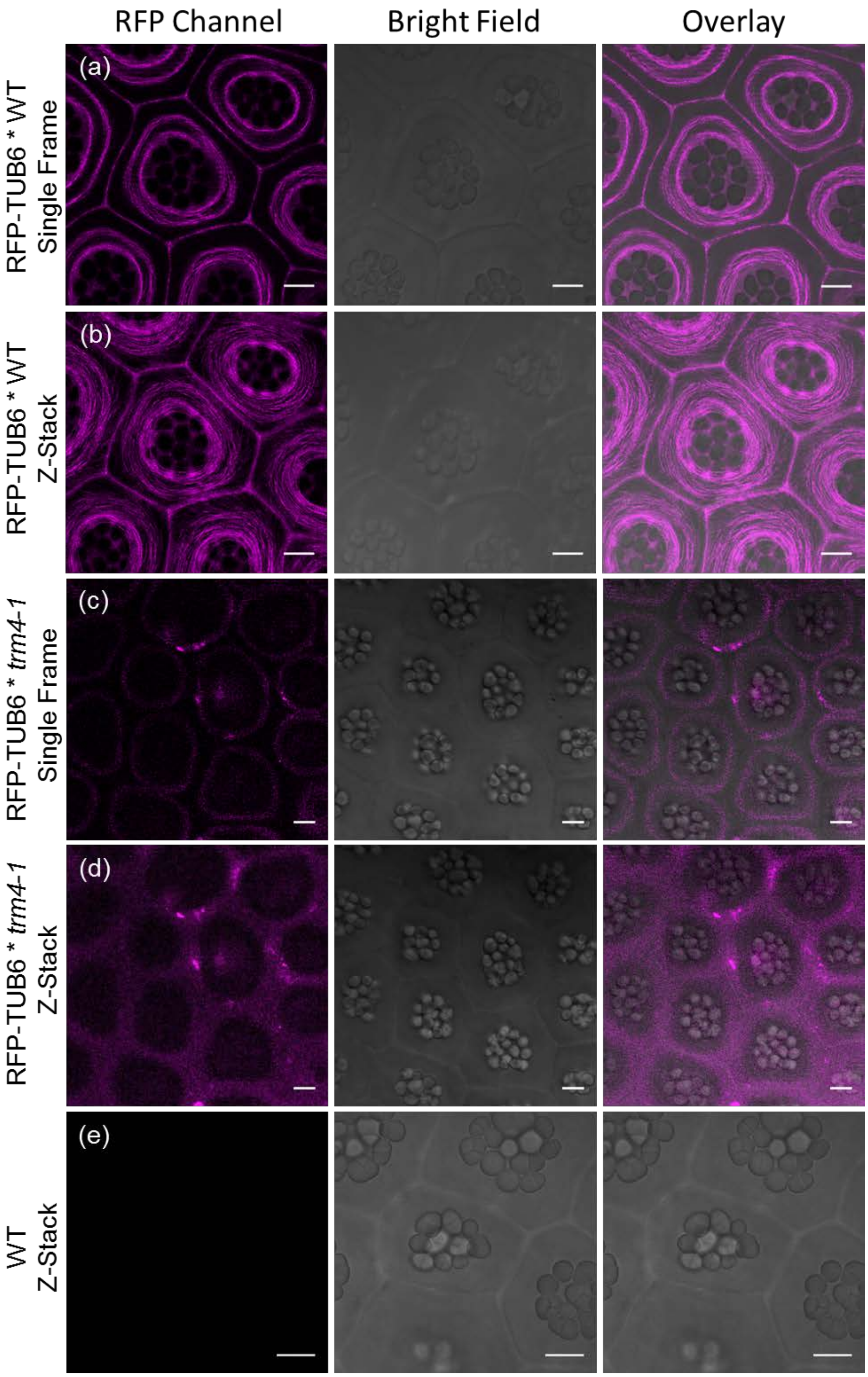

Fig. S14 Microtubule distribution in multiple SCE cells.

RFP-TUB6 * WT (a,b), RFP-TUB6 * *trm4-1* (c,d) and WT (e) at the stage of 7DPA. Bars = 10 μm.

Table S1. Primers used in this study

Primers set used in this study	
Primer name	Sequence
Primers for identification of T-DNA insertional lines	
trm4-1-lp	ATGGTTCGTGTCCAGTAGTCG
trm4-1-rp	TGGGATCAACACTGGAGTTTC
trm4-2-lp	GTACGTTGAAGCGTCGAGAAC
trm4-2-rp	AAGATCTGCGAGCCCTTAAAC
trm4-3-lp	CTTATCAAACGAGCTGGTACATGA
trm4-3-rp	GTTAGACAGAGCAGACGAGAGGAT
trm3-1-lp	GATGAACACGATGATGAGAAAGAA
trm3-1-rp	ATATGAAATATGGAAAGTGGCGTC
trm3-2-lp	AATCTCACCGTGCACAATCTC
trm3-2-rp	GAAACCGGTTTTCTCAAAAGC
cesa5-1-lp	TTGTGTTTTACCTCAGGGACG
cesa5-1-rp	TAATAACGTGCGAGATCACCC
muci10-1-lp	AAACCTACCAATCGAAACACG
muci10-1-rp	TAAACCAAACCAGACAATGCC
csla2-3-lp	TAGATGGTCTTGTGGACCTGC
csla2-3-rp	CAAAAGAACCCTTGGAGCTTC
o3144-GABI lines	GTGGATTGATGTGATATCTCC
LBb1.3-SALK lines	ATTTTGCCGATTTCGGAAC
Primers for TRM4 promoter cloning in pPLV13	
pPLV13-pTRM4-fp	tagttggaatgggttcgaaACTACGACGTACTACTATACGTACCTT
pPLV13-pTRM4-rp	ttatggagttgggttcgaaTTTCTACAGATCACAAAAACAATTCTCC
Primers for TRM4 promoter cloning in pCV01	
pCV01-pTRM4-*Kpn*I-fp	actgtcaaGGTACCactacgacgtactactatacgtacctt
pCV01-pTRM4-*Apa*I-rp	actgtcaaGGGCCCtttctacagatcacaaaaacaattctcc
Primers for TRM4 cds cloning in pCV01	
pCV01-TRM4 cds-fp	tagttggaataggttcATGGCTGCGAAGCTTCTGCATTCA

pCV01-TRM4 cds-rp	agtatggagttgggttcacCTGGTCAGCAAAAAGCGTTCGT

Primers for TRM4F1/2/3 cds cloning in pTRAkt

pTRAkt-EcoRI-TRM4F1-fp	actgtcaaGAATTCATGGCTGCGAAGCTTCTGCAT
pTRAkt-EcoRI-TRM4F1-rp	actgtcaaACATGTGCGGTGAGCTAGGAGAAAAACG
pTRAkt-EcoRI-TRM4F2-fp	actgtcaaGAATTCATGAGAAGCTTGGGGAAGGAC
pTRAkt-EcoRI-TRM4F2-rp	actgtcaaACATGTG AGCAGAGGCTTCTGTTTCGGA
pTRAkt-EcoRI-TRM4F3-fp	actgtcaaGAATTCATG TGTGTAGAAAAATCTACTGAA
pTRAkt-EcoRI-TRM4F3-rp	actgtcaaACATGTGCTGGTCAGCAAAAAGCGTTCG

Primers for TRM4 and TUB6 cds cloning in Gateway cassette

attB1-TRM4 cds	ggggacaagtttgtacaaaaaagcaggcttcGCTGCGAAGCTTCTGCATTCAT
attB2-TRM4 cds	ggggaccactttgtacaagaaagctgggtcCTACTGGTCAGCAAAAAGCGTT
attB1-TUB6 cds	ggggacaagtttgtacaaaaaagcaggcttcTCATGAGAGAAATCCTTCACATT
attB2-TUB6 cds	ggggaccactttgtacaagaaagctgggtcTCACTCATGATCCAATATCTC

Primers for qRT-PCR

UBQ10-qPCR-fp	CACACTCCACTTGGTCTTGCGT
UBQ10-qPCR-rp	TGGTCTTTCCGGTGAGAGTCTTCA
TRM4-qPCR-fp	CACCCGTGAAGACACAAGGTAATG
TRM4-qPCR-rp	ACTGTATGCTGGATTCCACTGGTC
TRM3-qPCR-fp	CCAGGAGGAAACATTTTGCAGAGG
TRM3-qPCR-rp	GCAGCCATGAATGGTGAAAAATAGC

Primers for MY2H and BIFC

CESA3-bifc/my2h-fp	GGGGACAAGTTTGTACAAAAAAGCAGGCTTCATGGAATCCGAAGGAGAAACC
CESA3-my2h-rp	GGGGACCACTTTGTACAAGAAAGCTGGGTCACAGTTGATTCCACATTCCAG
CESA3-bifc-rp	GGGGACCACTTTGTACAAGAAAGCTGGGTCTCAACAGTTGATTCCACATTC
TRM4-bifc/my2h-fp	GGGGACAAGTTTGTACAAAAAAGCAGGCTTCATGGCTGCGAAGCTTCTGCAT
TRM4-my2h-rp	GGGGACCACTTTGTACAAGAAAGCTGGGTCCTGGTCAGCAAAAAGCGTTCG
TRM4-bifc-rp	GGGGACCACTTTGTACAAGAAAGCTGGGTCCTACTGGTCAGCAAAAAGCGTT
CC1-bifc/my2h-fp	GGGGACAAGTTTGTACAAAAAAGCAGGCTTCATGCACGCCAAAACCGATTCC
CC1-my2h-rp	GGGGACCACTTTGTACAAGAAAGCTGGGTCAACTGTAGTGACGGTGCAATT
CC1-bifc-rp	GGGGACCACTTTGTACAAGAAAGCTGGGTCTCAAACTGTAGTGACGGTGCA
TRM26-bifc/my2h-fp	GGGGACAAGTTTGTACAAAAAAGCAGGCTTCATGGGAGGATTATTGCATCTCTTC
TRM26-my2h-rp	GGGGACCACTTTGTACAAGAAAGCTGGGTCACATATTTGTCTTCTTAGATCCTT
TRM26-bifc-rp	GGGGACCACTTTGTACAAGAAAGCTGGGTCTTAACATATTTGTCTTCTTAGATC

**Primers for sequencing**	
pPLV13-seq-fp	GTTGTAAAACGACGGCCAGT
pPLV13-seq-rp	CGCTGATCAATTCCACAGTT
pCV01-seq-fp	TGACGTAAGGGATGACGCA
pCV01-seq-rp	GCTGAACTTGTGGCCGTTTAC
pDONR201-seq-fp	TCGCGTTAACGCTAGCATGGATCTC
pDONR201-seq-rp	GTAACATCAGAGATTTTGAGACAC
pTRM4-seq-fp	GTGACCTCACCGGCAAATATT
TRM4 cds-seq-fp	AAGAGTTGCCTAGGCTTTCG
TRM4 cds-seq-rp	CTGGCGAAAAGCTTGATGTG
**Primers for confirmation of pPLV13 transformation**	
pPLV13-seq-fp1	GCGGGCCTCTTCGCTATTAC
pPLV13-seq-rp	CGCTGATCAATTCCACAGTT
**Primers for confirmation of pTRM4:GUS transformation**	
pTRM4-seq-fp	GTGACCTCACCGGCAAATATT
pPLV13-seq-rp	CGCTGATCAATTCCACAGTT
**Primers for confirmation of pTRM4:TRM4-sYFP transformation**	
pTRM4-seq-fp	GTGACCTCACCGGCAAATATT
pCV01-seq-rp	GCTGAACTTGTGGCCGTTTAC
**Primers for confirmation of P35SS:mYFP-TRM4 transformation**	
P35SS:mYFP-GWY-fp	CTGTCAGTTCCAAACGTAAAACGG
trm4-1-rp	TGGGATCAACACTGGAGTTTC

Table S2. Monosaccharide composition of non-adherent mucilage and total mucilage

Sugar content (μg mg^{-1} dry seed). Values represent the mean ± SD of four biological replicates. No significant difference of mucilage monosaccharide composition was found between the WT and *trm4* alleles.

	Non-Adherent Mucilage			Total Mucilage		
Sugar	WT	*trm4-1*	*trm4-2*	WT	*trm4-1*	*trm4-2*
Rha	9.50 ± 0.85	9.81 ± 0.69	9.79 ± 0.56	13.93 ± 0.82	14.02 ± 0.97	14.13 ± 0.74
Ara	0.20 ± 0.01	0.22 ± 0.01	0.19 ± 0.03	0.30 ± 0.01	0.31 ± 0.03	0.30 ± 0.01
Gal	0.29 ± 0.04	0.34 ± 0.04	0.33 ± 0.01	0.73 ± 0.06	0.73 ± 0.05	0.75 ± 0.04
Glc	0.16 ± 0.02	0.22 ± 0.04	0.24 ± 0.05	0.39 ± 0.10	0.36 ± 0.04	0.37 ± 0.03
Xyl	0.52 ± 0.05	0.51 ± 0.01	0.52 ± 0.01	0.87 ± 0.04	0.84 ± 0.02	0.86 ± 0.03
Man	0.12 ± 0.02	0.11 ± 0.01	0.13 ± 0.01	0.21 ± 0.01	0.20 ± 0.02	0.21 ± 0.01
GalA	9.82 ± 0.45	9.32 ± 0.94	9.90 ± 0.66	17.64 ± 1.35	17.65 ± 0.50	17.89 ± 1.36
Total	20.64 ± 0.83	20.55 ± 0.33	21.13 ± 0.28	34.12 ± 1.87	34.17 ± 1.12	34.55 ± 1.82

References

Drevensek S, Goussot M, Duroc Y, Christodoulidou A, Steyaert S, Schaefer E, Duvernois E, Grandjean O, Vantard M, Bouchez D, *et al.* 2012. The Arabidopsis TRM1-TON1 interaction reveals a recruitment network common to plant cortical microtubule arrays and eukaryotic centrosomes. *The Plant Cell* **24**: 178–191.
